# SLC11A2 withholds divalent metals from *Salmonella* in the gut epithelium

**DOI:** 10.1073/pnas.2532675123

**Published:** 2026-06-22

**Authors:** Emilia S. Norberg, Trina L. Westerman, Eddy Cruz, Summer D. Bushman, John Salogiannis, Eric P. Skaar, Johanna R. Elfenbein, Leigh A. Knodler

**Affiliations:** ^a^ Department of Microbiology and Molecular Genetics, Robert Larner, M.D. College of Medicine, University of Vermont, Burlington, VT 05405; ^b^ Department of Pathobiological Sciences, School of Veterinary Medicine, University of Wisconsin-Madison, Madison, WI 53706; ^c^ Department of Pathology, Microbiology, and Immunology, and Vanderbilt Institute for Infection, Immunology, and Inflammation, Vanderbilt University Medical Center, Nashville, TN 37232; ^d^ Department of Molecular Physiology and Biophysics, Robert Larner, M.D. College of Medicine, University of Vermont, Burlington, VT 05405; ^e^ Paul G. Allen School for Global Health, College of Veterinary Medicine, Washington State University, Pullman, WA 99164

**Keywords:** *Salmonella enterica*, intestinal epithelial cell, nutritional immunity, enteric infections, bacteria-containing vacuole

## Abstract

There is a constant tug-of-war for transition metals at the pathogen–host interface. Vertebrate hosts modulate the availability of metals to pathogens in a process known as nutritional immunity, but pathogens have evolved numerous countermeasures to this host defense strategy. The bioavailability of trace metals therefore shapes the outcome of disease. In mammals, epithelial cells lining the intestine are a major site of metal absorption. Intestinal epithelial cells (IECs) are also a target for invading enteric pathogens but the contribution of epithelium-intrinsic factors toward nutritional immunity is unclear. Using *Salmonella enterica* serovar Typhimurium (STm) harboring genetically encoded fluorescent sensors for transition metals, we mapped the spatiotemporal nature of metal competition during enteric salmonellosis. In contrast to the metal replete lumen, a subpopulation of STm experience a temporal, cell-specific restriction of Fe^2+^ and Zn^2+^ (≤0.1 µM), and possibly Mn^2+^, in both IECs and cells of the lamina propria during the early stages of infection. We further studied the contribution of the broad specificity divalent metal transporter, SLC11A2, in IECs to nutritional immunity against STm. SLC11A2 was recruited to maturing *Salmonella*-containing vacuoles and knockout of *SLC11A2* led to increased bacterial proliferation in IECs. Metal-responsive fluorescent reporters showed that vacuolar STm were less starved for Fe^2+^, and possibly Mn^2+^, but not Zn^2+^ or Mg^2+^ in the absence of *SLC11A2*. STm counters SLC11A2-mediated growth restriction in IECs via the Mn^2+^/Fe^2+^ transporter, MntH, and iron-binding siderophores. We conclude that SLC11A2-mediated sequestration of a subset of metals is an IEC innate defense mechanism against STm.

## Significance

There is limited source of trace metals in the gut for which invading pathogens and the infected host must compete. Using *Salmonella enterica* as a model enteric pathogen, along with fluorescent sensors of metal ion availability, we traced the sites of metal restriction in the bovine intestinal mucosa to intestinal epithelial cells (IECs) and phagocytes in the underlying lamina propria. We further show that SLC11A2 (NRAMP2), which is the sole SLC11 family member expressed in IECs and localizes to the apical membrane and endosomal network, limits the intracellular proliferation of *S. enterica* by withholding iron and manganese. Our findings show that nutrient limitation is a defense mechanism used by IECs against invading microbial pathogens.

Pathogens and their vertebrate hosts both require trace nutrient metals for cellular functions. To establish infection, pathogenic microorganisms actively scavenge trace nutrient metals from the extracellular space and host cells, plus possess mechanisms to minimize metal toxicity. As a counter-defense, the host can withhold transition metals to starve, or deliver metals in excess to intoxicate, microbial invaders. This concept is known as “nutritional immunity” (1). The solute carrier 11 (SLC11) or Natural resistance-associated macrophage protein (NRAMP) family sits at the nexus between host and microbe in the tug-of-war for metals. These transition metal transporters are conserved from bacteria to humans. In bacteria, SLC11/NRAMP proteins are represented by the proton-dependent manganese transporter (MntH) family. In vertebrates, there are two SLC11 paralogs—*SLC11A1* (also known as *NRAMP1*) and *SLC11A2* [also known as *NRAMP2*, divalent metal transporter 1 (*DMT1*) or divalent cation transporter 1 (*DCT1*)] in humans, and *Slc11a1* and *Slc11a2* in mice. These two transporters have different cellular and subcellular localizations (2) and substrate specificities (3–7).

In vivo studies have convincingly defined the importance of Slc11a1 in myeloid cells to nutritional immunity and, as a result, Slc11a1 has assumed a place of prominence in metal withholding during infection. *Slc11a1* was originally identified as a locus that confers natural resistance of some inbred mouse strains to **Mycobacterium*, Leishmania,* and *Salmonella* infection (8–10). These inbred mice have a naturally occurring missense mutation (G169D) in Slc11a1 (11, 12) and are characterized by the absence of the mature glycosylated protein form of the transporter (13). *Slc11a1*^G169D^ and *Slc11a1*^−/−^ mice are phenotypically indistinguishable (11). In humans, allelic variants of *SLC11A1* are associated with susceptibility to tuberculosis, leprosy, and leishmaniasis (14–16). *Slc11a1* expression is restricted to myeloid cells, e.g. macrophages, dendritic cells, and neutrophils (17–19), and Slc11a1 localizes to late endosomes and lysosomes (2). Slc11a1 depletes transition metals from phagolysosomes to control microbial growth (20–23). Slc11a1/SLC11A1 also increases proinflammatory antimicrobial immune responses via nitric oxide and lipocalin production, bactericidal activity of neutrophils, and activation of innate lymphocytes (24–26).

Unlike *Slc11a1*, *Slc11a2*/*SLC11A2* is ubiquitously expressed and particularly abundant in the proximal intestine, the primary site of trace metal absorption in the body [(6); The Human Protein Atlas; https://www.proteinatlas.org/ENSG00000110911-SLC11A2/tissue]. Slc11a2 was first identified as a divalent metal ion transporter in inbred mice (6, 27) and rats (28). Microcytic anemia (*mk/mk*) mice and Belgrade (*b/b*) rats are Fe^2+^ and Mn^2+^ deficient (3, 29, 30) due to a point mutation (G185R) in Slc11a2 which affects its stability and trafficking (31), and reduces its transport of Fe^2+^, Ni^2+^, and Mn^2+^ (29, 32, 33). The *mk/mk* mouse and *b/b* rat anemia phenotypes are, however, less severe than that of *Slc11a2*^−/−^ mice, which die soon after birth (34). *SLC11A2* mutations are extremely rare in humans; the few reported impact exon splicing (35, 36), or lead to point mutations or amino acid deletions (37–39) in SLC11A2. Patients exhibit microcytic anemia, and hepatic iron overload appears at a young age in some cases (35, 36, 38, 39). *SLC11A2* expression and/or SLC11A2 function have also been implicated in the pathogenesis of neurodegenerative diseases and inflammatory bowel disorders (40, 41). The impact of SLC11A2 on infection outcome is not known.

*Salmonella enterica* infection in humans presents as distinct clinical syndromes depending upon the serotype. Nontyphoidal *Salmonella* (NTS) serotypes, e.g., *S*. Typhimurium (STm) and *S*. Enteritidis, cause self-limiting diarrhea in immunocompetent individuals characterized by severe neutrophilic intestinal inflammation, or an invasive disease characterized by bacteremia in immunocompromised individuals. During typhoid fever, a systemic disease caused by human host restricted *S*. Typhi and *S*. Paratyphi, bacteria persist within professional phagocytes in granulomas in the liver, spleen, and bone marrow (42). Although the clinical syndromes differ between serotypes, the intestinal epithelium is the first line of host defense against these food-borne pathogens. Despite the presence of Slc11a1-positive cells in the intestinal lamina propria, and their enrichment during STm infection (19, 43), *Slc11a1* has little to no effect on STm replication in the mouse gut (43, 44). By contrast, the primary sites of Slc11a1-dependent control are the spleen, liver, and mesenteric lymph nodes, where STm primarily resides within macrophages (21, 43–45). Altogether, these observations raise the question of whether other SLC11 proteins also contribute to host defense. To close this knowledge gap, here we sought to define the relationship between SLC11A2, intestinal epithelial cells (IECs), and nutritional immunity during infection with the model enteric pathogen, STm. Using a natural host for NTS, the calf enteritis model (46), in combination with STm harboring metal ion responsive fluorescent reporters, we show that the mammalian host restricts Fe^2+^ and Zn^2+^, and possibly Mn^2+^, bioavailability in the intestinal mucosa, first in IECs, then in lamina propria phagocytes. Further, using CRISPR/Cas9 edited IECs we establish that SLC11A2 restricts Fe^2+^ and Mn^2+^ supply from intracellular STm, and limits STm proliferation. Our data support SLC11A2-mediated nutritional immunity as an intrinsic defense mechanism of the intestinal epithelium against invading pathogens.

## Results

### STm Is Starved of Trace Metals During the Acute Stages of Enteric Infection.

**Fig. 1.**
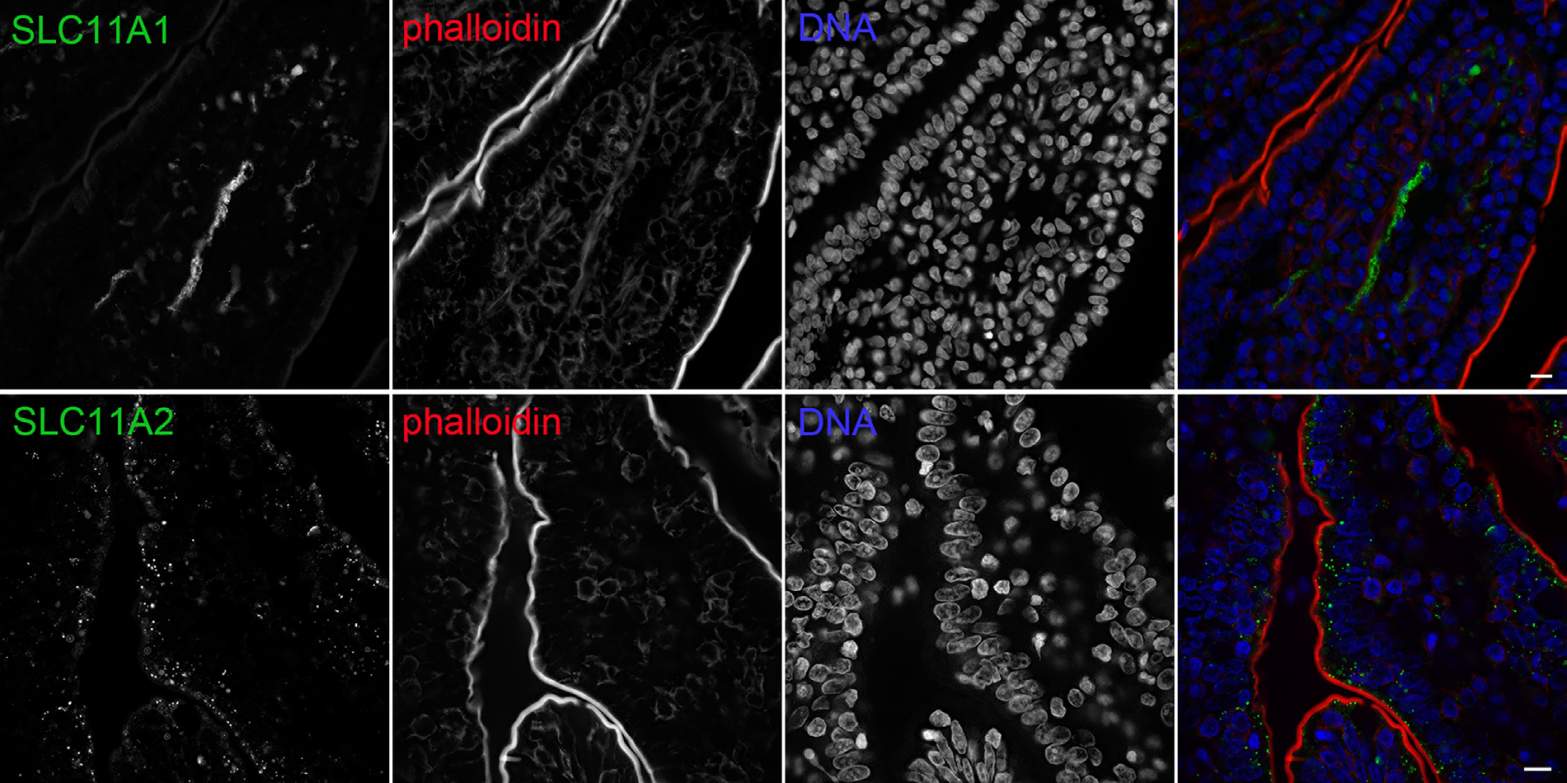
Spatially distinct localization of SLC11A1 and SLC11A2 in intestinal mucosa. Mock-infected (2 h postinoculation with PBS) bovine intestinal tissues were immunostained with polyclonal antibodies against SLC11A1 (NRAMP1, *Upper* panels) or SLC11A2 (NRAMP2, *Lower* panels). Phalloidin was used to visualize F-actin at the apical surface of epithelial cells. DNA was stained with DAPI. (Scale bar, 10 µm.)

The calf is a relevant animal model for studying acute *Salmonella*-induced enteritis because STm is a natural pathogen of cattle, STm infection causes similar signs of disease and pathology as observed in humans (46), and the *SLC11A1* G169D mutation is not found in European breeds of cattle (47). The ligated ileal-jejunal loop model in calves is especially suited for investigating *Salmonella*–host interactions in the gut at early times postinfection (p.i.) (46). To validate this model for the study of SLC11-mediated metal ion restriction during acute enteric infection, we immunolocalized SLC11A1 and SLC11A2 in uninfected intestinal tissue. SLC11A1 localized to discrete cells in the lamina propria and submucosa (Fig. 1), likely phagocytes and endothelial cells given that bovine *SLC11A1* is primarily expressed in these cell types (26, 48). SLC11A1 is similarly restricted to lamina propria phagocytes in the human colon (https://www.proteinatlas.org/ENSG00000018280-SLC11A1/tissue) and mouse small intestine (19, 43). By contrast, SLC11A2 staining was concentrated at the luminal surface of IECs as well as puncta within IECs in the calf ileum (Fig. 1), as observed in human duodenum (49, 50) and colon (https://www.proteinatlas.org/ENSG00000110911-SLC11A2/tissue). The distinct anatomic and subcellular distributions of these two metal transporters suggest that they play discrete roles in host defense in the mammalian gastrointestinal tract.

**Fig. 2.**
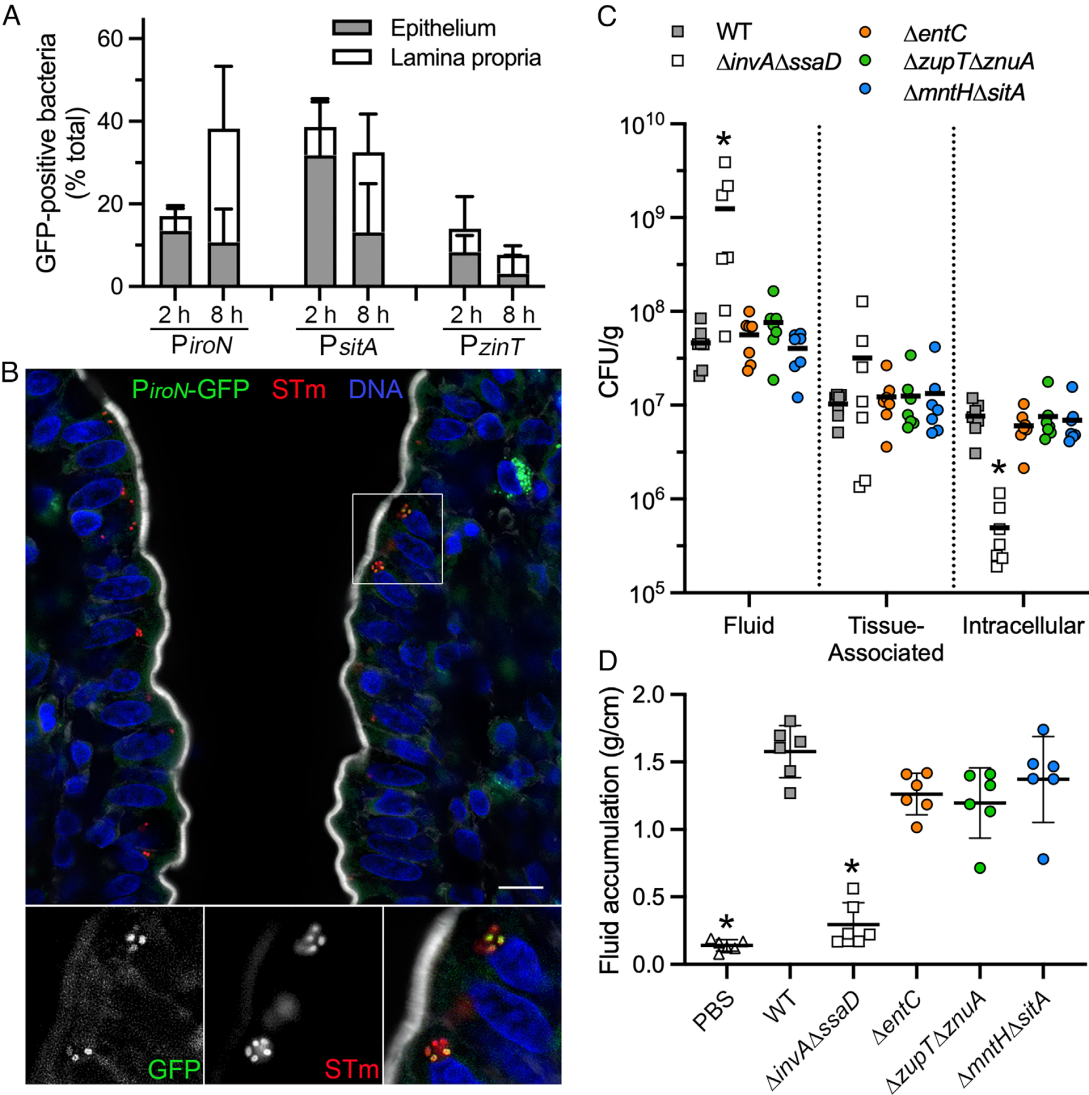
A subpopulation of *Salmonella* is deprived of iron, manganese, and zinc in the intestinal mucosa. (*A*) Ligated jejunal-ileal loops were inoculated with STm-mCherry harboring P*iroN*-*gfpmut3*, P*sitA*-*gfpmut3,* or P*zinT*-*gfpmut3* transcriptional reporters (~10^9^ CFUs). Tissues were collected at 2 h or 8 h postinoculation and fixed. Sections were stained with phalloidin to demarcate the apical surface of epithelial cells and Hoechst 33342 for DNA. Tissue-associated GFP-positive bacteria were scored within the epithelium (i.e., the single layer of cells lining the villi) or the lamina propria (i.e., in the tissue beneath the epithelium). Mean ± SD from n ≥ 4 calves. (*B*) Representative fluorescence microscopy image of intestinal tissue infected with STm-mCherry harboring a P*iroN*-*gfpmut3* reporter at 2 h postinoculation. GFP-positive bacteria are green, all *Salmonella* are red, DNA is blue and actin is grayscale. (Scale bar, 10 µm.) (*C* and *D*) Ligated ileal loops were inoculated with PBS, wild-type (WT) bacteria or the indicated deletion mutant (~10^9^ CFU) for 8 h. CFU from washed intestinal tissue (total tissue-associated bacteria), gentamicin-treated intestinal tissue (intracellular), and fluid were enumerated by serial dilution and plating on LB agar. (*C*) CFU were normalized to the tissue or fluid weight. (*D*) Fluid weight was normalized to loop length. Each symbol represents data from a single loop from one calf. *P < 0.05, significantly different from ST4/74 WT bacteria, ANOVA with Dunnett’s multiple comparisons test.

We next determined the spatiotemporal nature of metal ion availability during acute *Salmonella*-induced enteritis. We previously showed that STm carrying plasmids encoding *iroN*, *sitA,* or *zinT* promoters fused to *gfpmut3* serve as sensitive reporters of Fe^2+^, Fe^2+^/Mn^2+^, and Zn^2+^, respectively, in vitro and in cellulo (51). Expression of these genes (i.e., GFP fluorescence) is induced when Fe^2+^ (P*iroN*), Fe^2+^ and Mn^2+^ (P*sitA*), and Zn^2+^ (P*zinT*) concentrations are ≤0.1 µM (51). These genes should be lowly expressed in the gut lumen as iron, manganese, and zinc are present in the millimolar range (52–55). *iroN* encodes a receptor for enterobactin and salmochelin, two high-affinity Fe^3+^-binding siderophores (56, 57), the *sitABCD* operon encodes an ABC-type Mn^2+^ importer that belongs to the NRAMP superfamily (58) and *zinT* encodes an accessory protein for the high-affinity Zn^2+^ importer, ZnuABC (59). Bovine ileal loops were inoculated with wild-type bacteria constitutively expressing *mCherry* on the chromosome (STm-mCherry) and harboring P*iroN*-*gfpmut3*, P*sitA*-*gfpmut3,* or P*zinT*-*gfpmut3* reporters. Tissues were collected at 2 h or 8 h postinoculation, fixed, and sectioned. The infection times represent prior to (2 h), and after (8 h), severe neutrophilic inflammation (46). Tissue sections were stained with phalloidin to demarcate the epithelial brush border and DAPI for DNA (host cell nuclei). The number of tissue-associated GFP-positive STm-mCherry in the “epithelium” (i.e. bacteria in the single layer of cells lining the villi) or “lamina propria” (*i.e.* bacteria in the tissue beneath the epithelium) was scored by fluorescence microscopy (Fig. 2*A*). GFP-positive bacteria were detected as early as 2 h postinoculation—17.0 ± 7.0% for P*iroN*-*gfpmut3*, 38.7 ± 19.5% for P*sitA*-*gfpmut3* and 14.0 ± 6.7% for P*zinT*-*gfpmut3*—indicating that some, but not all, STm are exposed to ≤0.1 µM Fe^2+^ and Zn^2+^, and possibly Mn^2+^, shortly after internalization into the intestinal mucosa (Fig. 2*A*). GFP-positive bacteria were primarily within IECs at 2 h (Fig. 2 *A* and *B*). By 8 h postinoculation, 38.3 ± 11.8% of P*iroN-gfpmut3*, 32.5 ± 14.4% of P*sitA*-*gfpmut3*, and 7.6 ± 6.1% of P*zinT*-*gfpmut3* harboring bacteria were GFP-positive, mostly in the lamina propria (Fig. 2*A*). Therefore, in contrast to the metal-replete gut lumen, a subpopulation of STm is starved for trace metals (≤0.1 µM) in multiple cell types and at distinct anatomic sites during the early stages of enteric infection.

We tested the impact of metal limitation on bacterial survival in the calf intestine. We compared the fitness of wild-type bacteria with ∆*entC* (defective in Fe^3+^-binding siderophore synthesis, i.e., enterobactin and salmochelin), ∆*mntH*∆*sitA* (defective in Mn^2+^ >> Fe^2+^ import), and ∆*zupT*∆*znuA* (defective in Zn^2+^ >> Mn^2+^/Co^2+^ import) mutants at 2 h and 8 h postinoculation. Compared to wild-type bacteria, we observed no significant differences in bacterial burden for ∆*entC*, ∆*zupT*∆*znuA,* or ∆*mntH*∆*sitA* mutants in any tissue compartment (Fig. 2*C* and *SI* *Appendix*, Fig. S1); there was a small but not statistically significant reduction in fluid accumulation, a proxy for neutrophilic inflammation (60), in loops inoculated with STm mutants at 8 h (Fig. 2*D*). The noninvasive ∆*invA∆ssaD* mutant, which lacks structural components of type III secretion system 1 (T3SS-1, defective for tissue invasion and induction of neutrophilic inflammation) and T3SS-2 (defective for intracellular replication), induced very little fluid accumulation (Fig. 2*D*) and was severely attenuated for intracellular colonization (Fig. 2*C*), as expected (61, 62). These data indicate that STm mutants deficient in Mn^2+^, Fe^2+^, and Zn^2+^ import, and siderophore biosynthesis, are not overtly defective for intestinal colonization during the first 8 h of enteric disease in calves.

### *SLC11A2* Deletion Leads to Enhanced Bacterial Proliferation in IECs.

**Fig. 3.**
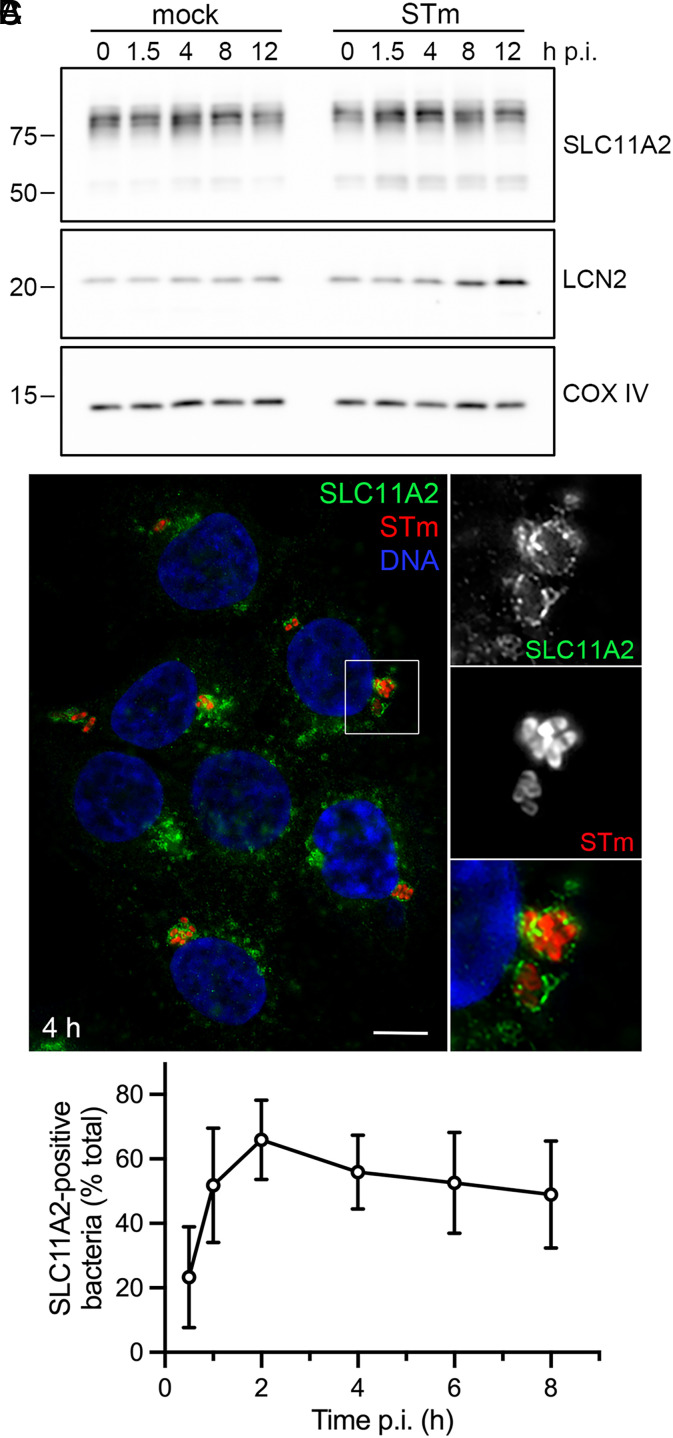
SLC11A2 is recruited to the *Salmonella*-containing vacuole. (*A*) HCT116 epithelial cells were mock-infected or infected with STm [McCoy’s media containing 1% (v/v) FCS]. Whole cell lysates were collected at the indicated times postinfection (p.i.). Proteins were separated by SDS-PAGE and subject to immunoblotting with antibodies against SLC11A2 (NRAMP2), lipocalin-2 (LCN2), a siderophore binding protein, and cytochrome c oxidase IV (COX IV). *LCN2* is induced in the intestinal mucosa following STm infection (74) and is a positive control. COX IV is a loading control. Molecular mass markers are indicated on the *Left*. Quantification of protein band intensity is shown in *SI* *Appendix*, Fig. S3. (*B* and *C*) HCT116 cells seeded on glass coverslips were infected with STm-mCherry [McCoy’s media containing 1% (v/v) FCS] and fixed with paraformaldehyde. Monolayers were permeabilized and immunostained with polyclonal antibodies against SLC11A2. DNA was stained with Hoechst 33342. (*B*) Representative fluorescence microscopy image shows SLC11A2 recruitment (green) to the vacuolar membrane surrounding STm-mCherry (red) at 4 h p.i. (Scale bar, 10 µm.) (*C*) Time course of SLC11A2 recruitment to the SCV. Samples were viewed by fluorescence microscopy and the proportion of SLC11A2-positive bacteria scored. Mean ± SD from n ≥ 3 independent experiments.

Given that SLC11A2 is highly expressed in IECs (Fig. 1), transports transition metals into mammalian cells (3–5, 7, 33), and STm experiences metal limitation in IECs in vivo (Fig. 2), we hypothesized that IEC-intrinsic SLC11A2 exerts modulatory effects on STm infection via nutritional immunity. We used HCT116 colonic epithelial cells as an infection model to test this hypothesis in cellulo. Fetal calf serum (FCS) is a rich source of trace metals (63) and iron-binding proteins, such as transferrin and ferritin (64), so we used tissue culture media (McCoy’s 5A) supplemented with 1% (v/v) FCS to minimize any saturation effects (*SI* *Appendix*, Fig. S2*A*). Immunoblotting of whole-cell lysates with anti-SLC11A2 polyclonal antibodies indicated multiple variants are present in HCT116 cells at ~50 kDa and ~80 kDa (Fig. 3*A*); alternative splicing generates multiple *SLC11A2* mRNA isoforms (33, 65). STm infection did not affect the level of any SLC11A2 variants over a 12 h time course (Fig. 3*A* and *SI* *Appendix*, Fig. S3*A*). Levels of SLC40A1 (ferroportin 1, FPN1), which exports Fe^2+^ and Mn^2+^ from IECs (66, 67), SLC39A14 (ZIP14), a Mn^2+^, Fe^2+^, Zn^2+^, and Cd^2+^ importer (68–72), CD71 (transferrin receptor 1, Tfr1), which mediates the uptake of transferrin-bound Fe^3+^ (73), and the cytosolic iron-binding protein, ferritin, were also unchanged during infection (*SI* *Appendix*, Fig. S3 *A* and *B*). By contrast, the amount of cellular lipocalin-2 (LCN2), a siderophore-binding protein, significantly increased (Fig. 3*A* and *SI* *Appendix*, Fig. S3*A*), which is consistent with the induction of *LCN2/Lcn2* in the intestinal mucosa upon STm infection in vivo (74).

SLC11A2 associates with the plasma membrane and endosomal network, particularly recycling endosomes, late endosomes, and lysosomes (2, 75–77). In uninfected HCT116 cells, SLC11A2 immunolocalized to punctate intracellular vesicles concentrated at the juxtanuclear region (Fig. 3*B*). Upon STm infection, SLC11A2 was temporally recruited to the *Salmonella*-containing vacuole (SCV) membrane (Fig. 3*B*). Specifically, there was a progressive increase in SLC11A2 decoration of the SCV up to 2 h p.i. (65.9 ± 12.3% SLC11A2-positive SCVs), followed by a plateau from 4-8 h p.i. (~50% SLC11A2-positive SCVs) (Fig. 3*C*). These acquisition kinetics are consistent with SCVs acquiring SLC11A2 as they mature along the endocytic pathway. Slc11a2 is also recruited to latex bead-containing phagosomes in mouse macrophages (75, 77).

To determine whether SLC11A2 exerts a protective effect against STm infection of IECs, HCT116 cells deficient in *SLC11A2* (*SLC11A2* KO) were generated by CRISPR/Cas9 technology. Immunoblotting confirmed the loss of multiple SLC11A2 variants in *SLC11A2* KO cells (*SI* *Appendix*, Fig. S4*A*). Deletion of *SLC11A2* did not have a compensatory effect on SLC40A1, SLC39A14, CD71, or LCN2 levels (*SI* *Appendix*, Fig. S4*A*). Ferritin plays an important role in cellular iron homeostasis by acting both as an iron-sequestering protein and a potential source of labile iron, and its synthesis and degradation are tightly regulated by the labile iron pool (78). *SLC11A2* KO cells had decreased amounts of ferritin heavy chain (FTH1) and ferritin light chain (FTL) (*SI* *Appendix*, Fig. S4*A*), suggesting reduced iron availability in the cytosol. Similar effects on ferritin levels have been reported for *SLC11A2* KO mouse embryonic fibroblasts (79) and human colonic epithelial cells (80). Because SLC11A2 is a H^+^/divalent metal symporter that localizes to endosomes, we also considered whether its absence affected endosomal acidification. Staining intensity and area for the acidotropic dye, LysoTracker Red DND-99, were equivalent for *SLC11A2* wild type (WT) and KO cells (*SI* *Appendix*, Fig. S4 *B* and *C*). As a positive control, WT cells were treated with bafilomycin A1, an inhibitor of the lysosomal H^+^-ATPase, which markedly decreased LysoTracker fluorescence (*SI* *Appendix*, Fig. S4 *B* and *C*).

**Fig. 4.**
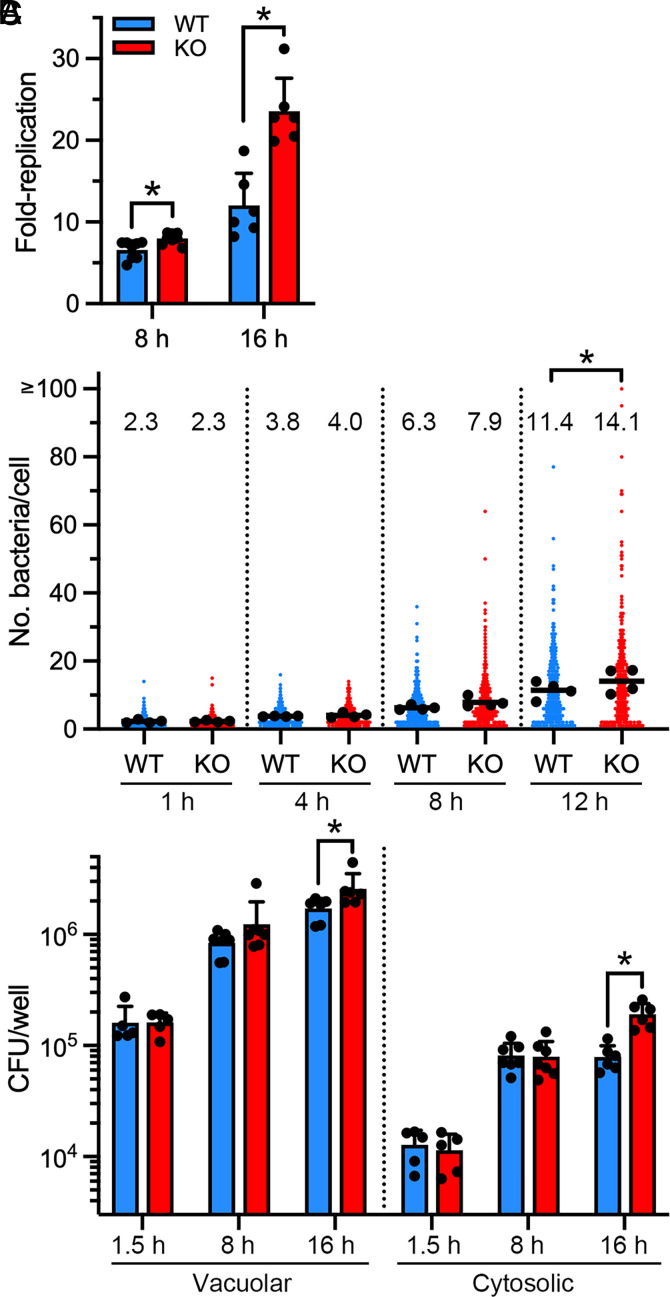
SLC11A2 limits *Salmonella* proliferation in IECs. (*A*) HCT116 *SLC11A2* WT and KO cells were infected with STm [McCoy’s media containing 1% (v/v) FCS] and the number of internalized bacteria at 1 h, 8 h, and 16 h p.i. quantified by gentamicin protection assay. Fold-replication was determined by dividing the 8 h p.i. and 16 h p.i. CFUs by those at 1 h p.i. Mean ± SD from at least 6 independent experiments. *P < 0.05, Student’s *t* test. (*B*) HCT116 *SLC11A2* WT and KO cells seeded on glass coverslips were infected with STm-mCherry. At 1 h, 4 h, 8 h, and 12 h p.i., monolayers were fixed and the number of bacteria per infected cell was blindly scored by fluorescence microscopy. Small dots represent individual cells; large dots the mean of each experiment; horizontal solid lines (and values at the top of each column) the average of 4 independent experiments. *P < 0.05, Student’s *t* test. (*C*) HCT116 *SLC11A2* WT and KO cells were infected with STm and the number of vacuolar and cytosolic bacteria at 1.5 h, 8 h, and 16 h p.i. was determined by CHQ resistance assay in conjunction with a gentamicin protection assay. Mean ± SD from at least 5 independent experiments. *P < 0.05, Student’s *t* test.

We compared the intracellular replication of STm in *SLC11A2* WT and KO cells using a gentamicin protection assay. Fold-replication was determined by dividing the colony-forming units (CFUs) at 8 h or 16 h p.i. by those at 1 h p.i. While there was no effect on invasion efficiency at 1 h p.i. (109,000 ± 24,900 CFU/well for WT; 103,125 ± 16,814 CFU/well for KO; n = 8), there was a significant increase in bacterial replication in *SLC11A2* KO cells compared to WT cells at 8 h (8.0 ± 0.7-fold versus 6.6 ± 1.1-fold, respectively; *P* < 0.05, Student’s *t* test) and 16 h p.i. (23.6 ± 4.1-fold versus 12.1 ± 3.9-fold, respectively; *P* < 0.05, Student’s *t* test; Fig. 4*A*) in McCoy’s media containing 1% (v/v) FCS. Replication over 16 h was also significantly increased for *SLC11A2* KO cells in McCoy’s media containing 5% (v/v) FCS (47.9 ± 12.1-fold versus 29.8 ± 1.8-fold, respectively; *P* < 0.05, Student’s *t* test; *SI* *Appendix*, Fig. S5*A*) but not 10% (v/v) FCS (65.9 ± 15.7-fold versus 53.7 ± 10.6-fold, respectively; *P* = 0.19, Student’s *t* test; *SI* *Appendix*, Fig. S6*A*). The increased proliferation was independently confirmed by scoring the number of STm-mCherry bacteria per cell by fluorescence microscopy. The mean number of bacteria per cell was comparable in WT and KO cells at 1 h and 4 h p.i. (Fig. 4*B*) but was increased in KO cells at 8 h p.i. and 12 h p.i. (Fig. 4*B*). STm occupies both vacuolar and cytosolic niches in epithelial cells (81). These populations can be distinguished using a chloroquine (CHQ) resistance assay in conjunction with a gentamicin protection assay, i.e., CHQ-resistant bacteria are cytosolic whereas CHQ-sensitive bacteria (total minus CHQ-resistant) are vacuolar (82, 83). There was no significant difference in the number of vacuolar or cytosolic bacteria at 1.5 h p.i. comparing *SLC11A2* WT and KO cells (Fig. 4*C*). At 8 h p.i., cytosolic CFUs were equivalent but vacuolar CFUs were increased by 1.47-fold in *SLC11A2* KO cells, albeit without statistical significance (*P* = 0.14, Student’s *t* test). By 16 h p.i., there was a significant increase in vacuolar and cytosolic populations in *SLC11A2* KO cells in 1% (v/v) FCS (1.51-fold and 2.43-fold, respectively; *P* < 0.05, Student’s *t* test; Fig. 4*C*) and 5% (v/v) FCS infection conditions (1.56-fold and 2.01-fold, respectively; *P* < 0.05, Student’s *t* test; *SI* *Appendix*, Fig. S5*B*), indicating that STm replication is enhanced in the absence of SLC11A2 in both intracellular niches.

### SLC11A2 Withholds Iron and Manganese From Intracellular STm.

The presence of Slc11a1 alters the expression of *Salmonella* pathogenicity island 2 (SPI2)-associated genes (*ssrA*, *sseA,* and *sseJ*) but not SPI-1 associated genes (*hilA*) or *phoP* in mouse macrophages (84, 85). We did not detect any overt difference in the temporal expression of SPI2- (*ssaG*) or SPI1-associated (*prgH*) genes in *SLC11A2* WT or KO epithelial cells (*SI* *Appendix*, Fig. S7*A*), discounting altered virulence gene expression as a likely reason for the increased bacterial replication. The kinetics of lysosomal-associated membrane protein 1 (LAMP1) acquisition, a hallmark of SCV maturation (86), were also comparable for WT and KO cells (*SI* *Appendix*, Fig. S7*B*). Instead, we hypothesized that SLC11A2 restricts intracellular STm replication by withholding metals. There is no clear consensus as to which metals are transported by SLC11A2. From transport studies with brush-border membrane vesicles, *Xenopus* oocytes and HEK293 cells, the substrate specificity of SLC11A2 is broad, i.e., Fe^2+^, Co^2+^, Mn^2+^, Ni^2+^, Pb^2+^, Cd^2+^, Cu^2+^, and Zn^2+^ (3–5, 7, 33). Therefore, we used an unbiased approach to compare total metal levels in *SLC11A2* WT and KO cells. Mock-infected and STm-infected HCT116 cells were collected at 8 h p.i. and metal ion concentrations were measured in cellular homogenates by inductively coupled plasma mass spectrometry (ICP-MS). ^34^S was used as a reference element for normalization across samples (87). Magnesium was the most abundant metal and cobalt and nickel the least abundant in HCT116 cells (*SI* *Appendix*, Fig. S8). Comparing mock-infected *SLC11A2* WT and KO cells, total cellular iron levels trended lower in *SLC11A2* KO cells ([^56^Fe]/[^34^S] of 2.11x10^-3^ ± 1.50x10^-4^ for WT, 1.91x10^-3^ ± 1.24x10^-4^ for KO; *P* = 0.169, ANOVA with Tukey’s multiple comparisons test; *SI* *Appendix*, Fig. S8). The comparative abundance of other metals—^55^Mn, ^59^Co, ^60^Ni, ^66^Zn, ^63^Cu, and ^24^Mg—was unchanged in mock-infected cells (*SI* *Appendix*, Fig. S8). STm infection of WT cells caused a small but significant reduction in the level of cellular magnesium ([^24^Mg]/[^34^S] of 0.154 ± 0.0017 for mock, 0.150 ± 0.00038 for infected; *P* < 0.05, ANOVA with Tukey’s multiple comparisons test; *SI* *Appendix*, Fig. S8). For *SLC11A2* KO cells, infection did not significantly alter the total cellular level of any metal tested (*SI* *Appendix*, Fig. S8). Our unbiased quantitative analysis hints that a subset of metals is altered in SLC11A2 KO cells compared to WT cells under basal and infection conditions.

**Fig. 5.**
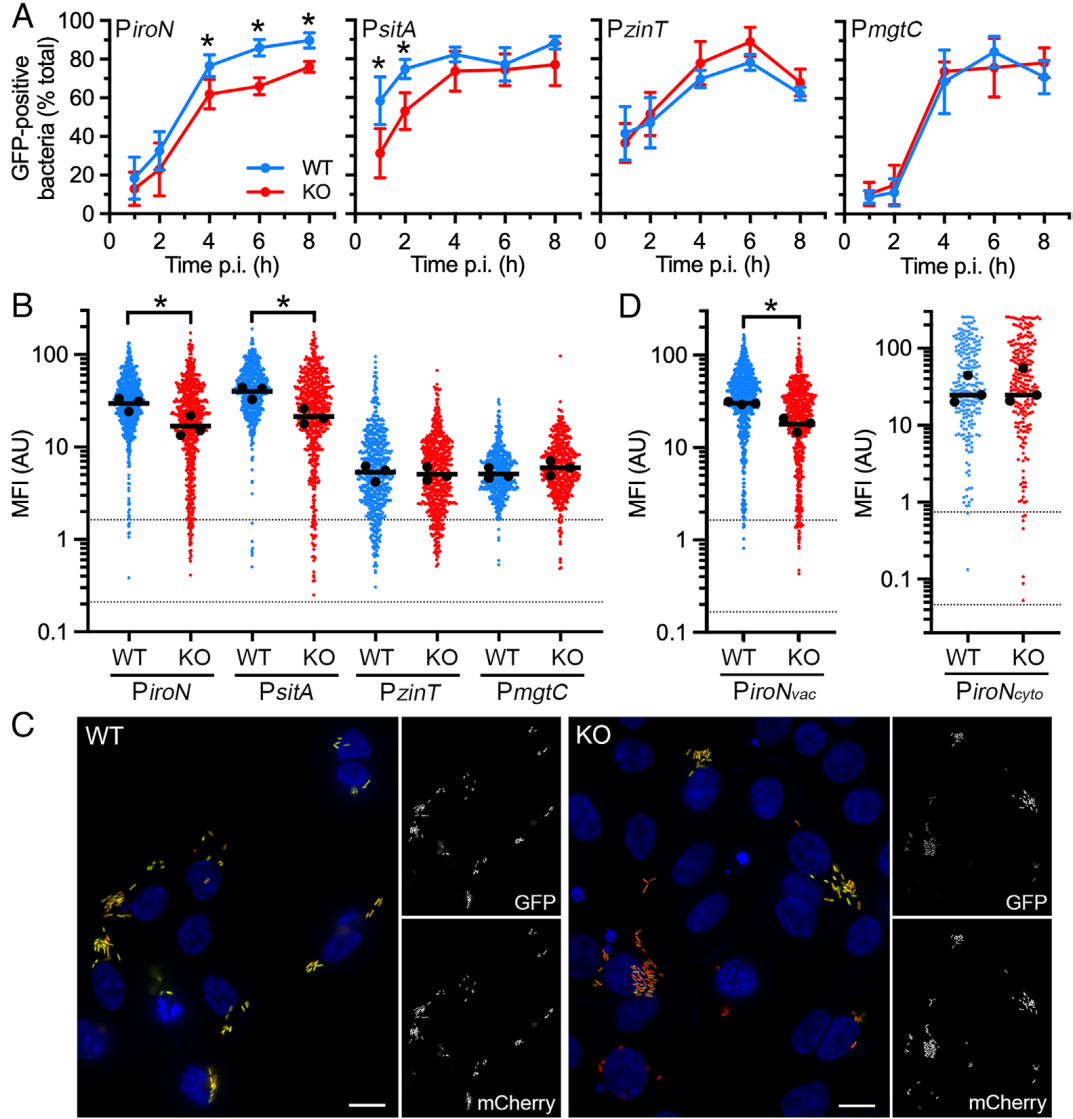
SLC11A2 withholds Fe^2+^ and Mn^2+^ from intracellular *Salmonella*. (*A*) HCT116 *SLC11A2* WT and KO cells seeded on glass coverslips [McCoy’s media containing 1% (v/v) FCS] were infected with STm-mCherry harboring the following fluorescent transcriptional reporters: P*iroN*-*gfpmut3*, P*sitA*-*gfpmut3*, P*zinT*-*gfpmut3*, P*mgtC*-*gfpmut3*. At the indicated times, monolayers were fixed and stained with Hoechst 33342 to label DNA. The number of GFP-positive bacteria was scored by fluorescence microscopy. Mean ± SD from 3 independent experiments. *P < 0.05, Student’s *t* test. (*B*) Quantification of the MFI of GFP signal by fluorescence microscopy and ImageJ analysis. Acquisition parameters (exposure time and gain) were the same for all reporters. AU, arbitrary units. Small dots represent individual bacteria; large dots indicate the geometric mean of each experiment; horizontal solid lines indicate the mean of three independent experiments. The dashed lines indicate the range of background fluorescence in the GFP channel for mCherry-STm (no reporter plasmid). *P < 0.05, Student’s *t* test. (*C*) Representative images of HCT116 *SLC11A2* WT and KO cells infected with STm-mCherry P*sitA*-*gfpmut3* bacteria at 8 h p.i. (Scale bar, 10 µm.) (*D*) HCT116 *SLC11A2* WT and KO cells seeded on glass coverslips were infected with STm harboring P*iroN*-*gfpmut3* and a low copy reporter plasmid for vacuolar (P*ssaG*-*mCherry*) or cytosolic (P*uhpT*-*mCherry*) residence. Monolayers were fixed at 8 h p.i. and the MFI of GFP signal in mCherry-positive bacteria was quantified by fluorescence microscopy and ImageJ analysis. The *Left* panel is vacuolar STm (P*iroN*_vac_); the *Right* panel is cytosolic STm (P*iroN*_cyto_). *P < 0.05, Student’s *t* test.

To complement the population-based ICP-MS analysis, we used STm as a fluorescent biosensor to report on intracellular metal concentrations at the single-cell level. HCT116 *SLC11A2* WT and KO cells were infected with STm-mCherry harboring metal-responsive fluorescent reporters (P*iroN*-*gfpmut3*, P*sitA*-*gfpmut3*, P*zinT*-*gfpmut3,* or P*mgtC*-*gfpmut3*), monolayers were fixed at various times p.i. and the number of GFP-positive bacteria was scored by fluorescence microscopy. For all four reporters, there was a temporal increase in the proportion of GFP-positive bacteria, indicative of transcriptional induction upon bacterial internalization (Fig. 5*A* and *SI* *Appendix*, Figs. S5*C* and S6*C*). A general trend was that more STm-mCherry harboring P*iroN*-*gfpmut3* and P*sitA*-*gfpmut3* were GFP-positive in *SLC11A2* WT compared to KO cells over time (Fig. 5*A* and *SI* *Appendix*, Figs. S5*C* and S6*C*), suggesting decreased Fe^2+^/Mn^2+^ availability in the presence of SLC11A2. STm-mCherry harboring P*zinT*-*gfpmut3* or P*mgtC*-*gfpmut3* reporters had comparable induction kinetics in WT and KO cells (Fig. 5*A* and *SI* *Appendix*, Figs. S5*C* and S6*C*).

We also considered heterogeneity in gene promoter-*gfp* expression in individual bacteria by quantifying the mean fluorescence intensity (MFI) using ImageJ. At 8 h p.i., the average MFI for bacteria harboring P*zinT*-*gfpmut3* and P*mgtC*-*gfpmut3* was unchanged in HCT116 *SLC11A2* WT and KO cells in McCoy’s media containing 1-10% (v/v) FCS (Fig. 5*B* and *SI* *Appendix*, Figs. S5*D* and S6*D*). By contrast, the average MFI for bacteria harboring P*iroN*-*gfpmut3* and P*sitA*-*gfpmut3* was significantly higher in WT cells for all FCS concentrations (Fig. 5 *B* and *C* (1% FCS), 1.77-fold for P*iroN*, 1.86-fold for P*sitA*; *P* < 0.05, Student’s *t* test; *SI* *Appendix*, Fig. S5*D* (5% FCS), 2.34-fold for P*iroN*, 2.85-fold for P*sitA*; *P* < 0.05, Student’s *t* test; *SI* *Appendix*, Fig. S6*D* (10% FCS), 2.93-fold for P*iroN*, 2.35-fold for P*sitA*; *P* < 0.05, Student’s *t* test). A similar trend was observed at 4 h and 12 h p.i. (*SI* *Appendix*, Fig. S9). Notably, the strength of *iroN* and *sitA* promoter induction (i.e., average MFI) depended on FCS concentration in the growth media, with the highest promoter activity in 1% (v/v) FCS (Fig. 5*B* and *SI* *Appendix*, Figs. S2*B*, S5*D*, and S6*D*). Furthermore, more bacteria were GFP-positive in 1% (v/v) FCS (Fig. 5*A* and *SI* *Appendix*, Figs. S5*C* and S6*C*). By contrast, the intensity and kinetics of *zinT* and *mgtC* induction were unaffected by FCS concentration (Fig. 5*A* and *SI* *Appendix*, Figs. S2*B*, S5*C*, and S6*C*) despite altered zinc and magnesium abundance in tissue culture media (*SI* *Appendix*, Fig. S2*A*).

In HCT116 cells, ≤10% of intracellular bacteria are cytosolic (Fig. 4*C*) compared to up to 50% in HeLa epithelial cells at later times p.i. (51), implicating vacuolar STm as those most likely to be experiencing reduced Fe^2+^/Mn^2+^ availability in *SLC11A2* WT cells (Fig. 5*B*). We used plasmid-based reporters of vacuolar (P*ssaG-mCherry*) and cytosolic (P*uhpT-mCherry*) residence, in conjunction with P*iroN*-*gfpmut3*, to pinpoint which intracellular population was impacted. The average MFI of GFP fluorescence was increased in *SLC11A2* WT cells for vacuolar bacteria only (Fig. 5*D*). Altogether, the increased expression of *iroN* and *sitA* reporters at the population and single-cell levels in WT cells, together with the ICP-MS analysis, points to SLC11A2 withholding Fe^2+^/Mn^2+^ from intracellular STm, primarily in the SCV. Additionally, the *zinT* and *mgtC* biosensors suggest that intracellular STm are exposed to similar levels of Zn^2+^ and Mg^2+^ in *SLC11A2* WT and KO cells.

### MntH and EntC Compete Against SLC11A2 for Trace Metals.

**Fig. 6.**
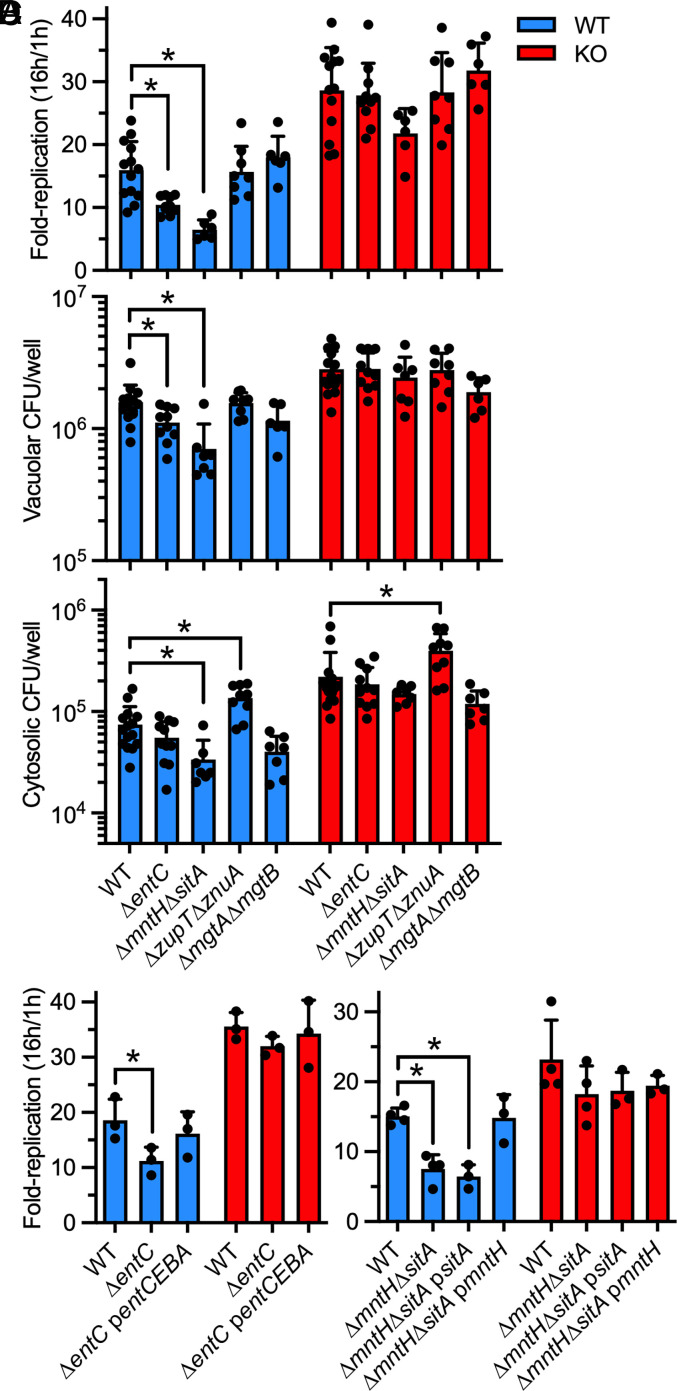
Iron and manganese acquisition by *Salmonella* counter SLC11A2 activity. (*A*) HCT116 *SLC11A2* WT and KO cells [McCoy’s media containing 1% (v/v) FCS] were infected with STm wild-type (WT), ∆*entC*, ∆*mntH*∆*sitA*, ∆*zupT*∆*znuA,* or ∆*mgtA*∆*mgtB* bacteria. Fold-replication was determined by gentamicin protection assay by dividing CFUs at 16 h by those at 1 h p.i. (*B* and *C*) The number of vacuolar (*B*) and cytosolic (*C*) bacteria at 16 h p.i. was determined by CHQ resistance assay in conjunction with a gentamicin protection assay. Mean ± SD from ≥6 independent experiments. *P < 0.05, ANOVA with Dunnett’s multiple comparisons test. (*D* and *E*) *In trans* complementation. (*D*) HCT116 SLC11A2 WT and KO cells were infected with STm wild-type (WT), ∆*entC* and ∆*entC* pWSK29-*entCEBA* bacteria or (*E*) STm wild-type (WT), ∆*mntH*∆*sitA*, ∆*mntH*∆*sitA* pWSK29-*sitA,* and ∆*mntH*∆*sitA* pWSK29-*mntH* bacteria. Fold-replication was determined by gentamicin protection assay by dividing 16 h p.i. CFUs by those at 1 h p.i. Mean ± SD from at least 3 independent experiments. *P < 0.05, ANOVA with Dunnett’s multiple comparisons test.

We next investigated whether bacteria deficient in metal acquisition were attenuated for growth in IECs, and if so, whether deletion of *SLC11A2* would rescue this attenuation. *SLC11A2* WT cells were infected with STm wild-type or mutants defective in high affinity Mn^2+^, low affinity Fe^2+^ import (∆*sitA*∆*mntH*), high affinity Zn^2+^, low affinity Mn^2+^/Co^2+^ import (∆*zupT*∆*znuA*), Mg^2+^ import (∆*mgtA*∆*mgtB*), or siderophore synthesis (∆*entC*). Growth over 16 h was quantified by gentamicin protection assay. Compared to wild-type bacteria (15.9 ± 4.6-fold), replication of ∆*entC* (10.4 ± 1.4-fold) and ∆*mntH*∆*sitA* (6.5 ± 1.5-fold) bacteria were significantly decreased in McCoy’s media containing 1% (v/v) FCS (Fig. 6*A*). Via CHQ resistance assay, ∆*entC* bacteria were replication-deficient in the SCV, whereas ∆*mntH*∆*sitA* bacteria were replication-deficient in both the SCV and cytosol (Fig. 6 *B* and *C*). Plasmid-borne complementation with *entC* (Fig. 6*D*) and *mntH*, but not *sitA* (Fig. 6*E*), rescued the replication defect of the respective deletion mutants in *SLC11A2* WT cells. The lack of *in trans* complementation suggests that the SLC11A2-dependent restriction of ∆*mntH*∆*sitA* bacteria is primarily due to the loss of MntH rather than SitA. Of note, MntH and SitABCD transport capacities have different pH optima; in acidic conditions (such as those found in the mature SCV lumen), MntH uptake of Mn^2+^ is maximal whereas SitABCD is largely inactive (58). The fold-replication for ∆*zupT*∆*znuA* and ∆*mgtA*∆*mgtB* mutants was comparable to wild-type bacteria in *SLC11A2* WT cells (Fig. 6*A*). In *SLC11A2* KO cells, replication of all the deletion strains was indistinguishable from wild-type bacteria (Fig. 6*A*). The ∆*zupT*∆*znuA* mutant replicated better than wild-type bacteria in the cytosol of WT and KO cells (Fig. 6*C*), indicating that this phenotype is SLC11A2-independent. We reason that deletion of SLC11A2 rescues the replication defect of ∆*entC* and ∆*mntH*∆*sitA* bacteria because it removes the host-advantage in the competition for Fe^2+^ and Mn^2+^, which directly connects the bioavailability of these two transition metals with SLC11A2-mediated restriction of STm growth in IECs.

## Discussion

STm face stiff competition for metal ions whether extracellular and intracellular, and at both intestinal and extraintestinal sites. Studies on metal ion competition between STm and its mammalian host have largely used C57BL/6 (*Slc11a1*^G169D^, i.e. *Slc11a1* null background) mice and streptomycin pretreatment to alter the resident gut microbiota. In this mouse colitis model, STm efficiently colonizes the cecum [though most bacteria remain extracellular (88)], and elicit severe neutrophilic inflammation and mild secretory responses in the cecum and proximal colon, then systemically spread to the liver and spleen ~48 h after inoculation (89). This infection model has defined much of what we know about the cell- and tissue-specificity of nutritional immunity against *S. enterica*. Metal levels in the intestinal lumen are in the millimolar range (52–55). In response to STm infection, neutrophils and other inflammatory cells release calprotectin, a metal chelator (53), leading to increased fecal calprotectin (a proxy for luminal content), and reduced fecal Zn^2+^ and Mn^2+^ (90). Likewise, increased Lcn2 production in the intestinal mucosa and secretion into the lumen promotes the sequestration of enterobactin produced by STm and other enteric bacteria (89). A small peptide hormone, hepcidin, is also released from hepatocytes to deplete extracellular iron (86–88). As a result, serum and fecal iron levels decline markedly in STm-infected mice (55, 90, 91). Decreased intracellular Fe^3+^ has also been reported in duodenal enterocytes during long-term STm infection of Sv129S6 (*Slc11a1*^+/+^) mice (90). Here, we show in a calf intestinal loop model that a subpopulation of intracellular STm is exposed to ≤0.1 µM Fe^2+^, Zn^2+^, and likely Mn^2+^, within IECs and lamina propria phagocytes during acute infection in vivo (Fig. 2).

The SLC11 transporters appear to play site- and cell-specific roles during infection. *Slc11a1* expression is ~50-fold higher than that of *Slc11a2* in phagocytes (75), suggesting that Slc11a1 function dominates in this cell type. In concordance, Slc11a1 limits STm replication in the spleen, liver, and mesenteric lymph nodes of mice (21, 43, 44), organs in which STm primarily resides within macrophages, but has little to no effect on STm replication in the mouse gut (43, 44). Mice deficient in *Slc11a2* die within 7 d of birth (34), which has limited the study of its contribution to host defense. *Slc11a2*^−/−^ bone marrow–derived macrophages supported increased STm burdens (92) with the caveat macrophages derived from C57BL/6 mice were used, which have a nonfunctional Slc11a1. Here, we have shown that SLC11A2 removes Fe^2+^/Mn^2+^ from bacteria-containing vacuoles in IECs in cellulo. Further work is needed to determine the impact of SLC11A2-mediated metal restriction on STm infection dynamics in vivo.

*S. enterica* employs numerous strategies to fight back in the war for metals. With regard to iron, STm produces a second siderophore, salmochelin, a glucosylated version of enterobactin that is not bound by LCN2 (93). An *iroN* (receptor for ferric-bound enterobactin and salmochelin) deletion mutant is defective for cecal colonization in streptomycin-pretreated mice at 48 h p.i. but not in the absence of intestinal inflammation (74). In a transposon screen for STm mutants defective for intestinal colonization in calves, pigs, and chickens, enterobactin and salmochelin synthesis mutants showed variable degrees of fitness depending on the Tn7 insertion site (94). Our study of STm and metal limitation is focused on intestinal tissue and cellular colonization, which distinguishes it from other studies which largely interrogate STm fitness in luminal contents (53, 54, 74, 95). The observed iron restriction (Fig. 2) did not translate to a colonization defect for the ∆*entC* mutant in the intestinal lumen or mucosa at 2 h (*SI* *Appendix*, Fig. S1) or 8 h (Fig. 2), in agreement with an earlier study of iron acquisition mutants (*iroN*; *tonB*, Fe^3+^ uptake; *feoB*, Fe^2+^ uptake) in bovine ligated loops (96). STm infection of ligated intestinal loops models the very early interactions between bacteria and the intestinal mucosa while avoiding bottlenecks associated with traversal of the stomach, duodenum, and proximal jejunum. An intrinsic limitation of this infection model is the limited number of bacterial doublings in the intestinal lumen over the short duration of the experiment (typically 8 h). STm deletion mutants are grown in rich broth prior to loop inoculation and do not have a growth defect in this medium (51, 97, 98). Bacteria might be sufficiently metal replete to allow them to survive and/or undergo limited replication in the face of the host inflammatory response in intestinal loops. Additionally, STm siderophore synthesis mutants can still acquire iron through other mechanisms in vivo such as Fe^3+^-bound xenosiderophores produced by resident microbiota (99, 100). Despite these caveats, it is clear that siderophore-mediated iron acquisition contributes to STm fitness at systemic sites (101), and at later times in the inflamed intestine (74) when bacteria are replicating and high-affinity iron binding proteins such as LCN2 and hepcidin have been released by the host (89).

STm can circumvent calprotectin-mediated metal limitation in the mouse gut through the expression of a high-affinity Zn^2+^ importer, ZnuABC (53), and two high-affinity Mn^2+^/low-affinity Fe^2+^ importers, MntH and SitA (54). Here, we identified transcriptional signatures of Zn^2+^, Fe^2+^ (and possibly Mn^2+^) limitation in bovine intestinal mucosa but we saw no difference in luminal or tissue colonization between wild-type and ∆*mntH*∆*sitA* bacteria at 2 h (*SI* *Appendix*, Fig. S1) or 8 h (Fig. 2*C*). Similarly, STm mutants defective for Mn^2+^ transport (∆*mntH*, ∆*sitA*, ∆*mntH*∆*sitA*) colonize the mouse cecum at levels equivalent to wild-type bacteria at 96 h p.i. (54). The lack of an overt phenotype might be explained by ∆*mntH*∆*sitA* mutants being able to acquire trace amounts of Mn^2+^ via ZupT and other unidentified mechanisms (102, 103), the metal-replete growth conditions prior to loop inoculation and/or the millimolar levels of metals in the gut lumen (52–55). In mixed infections, wild-type bacteria outcompete *mntH*, *sitA,* and *mntH sitA* deletion mutants in the small intestine and spleen of C57BL/6, BALB/C (i.e., *Slc11a1*^−/−^) and C3H/HeN (i.e., *Slc11a1*^+/+^) mice (54, 104), indicating that Mn^2+^ acquisition does provide STm with a competitive advantage at intestinal and extraintestinal sites. A Zn^2+^ uptake mutant is severely compromised in the mouse colitis model—cecal colonization by a ∆*znuA* mutant is reduced ~200-fold at 96 h p.i. (54)—but neither the ZnuABC transporter nor the IroN receptor provide a growth advantage in the mouse in the absence of gut inflammation (54), or in the bovine loop model (Fig. 2) (96).

We observed phenotypic heterogeneity in metal deprivation in cellulo and in vivo. Activation of *iroN*, *sitA*, *zinT,* and *mgtC* promoters was observed in the majority of STm by 2 to 4 h p.i. in cellulo but with a great degree of transcriptional heterogeneity (i.e., GFP intensity) (Fig. 5 and *SI* *Appendix*, Figs. S5 and S6), even within the same cell (Fig. 5). In vivo, the minority of STm experience metal limitation within IECs and cells of the lamina propria during acute enteric infection (Fig. 2). Similar results have been reported for STm and iron availability at systemic sites in C57BL/6 and BALB/c mice (45). Why most bacteria in cellulo and the minority in vivo? Cell culture media is surely a poor mimic of the in vivo extracellular environment, especially the gut lumen, which could substantially impact bacterial gene expression. Furthermore, the rate and extent of bacterial replication, and higher proportion of infected host cells in cellulo, might deplete available metals faster than in vivo. We also observed interbacterial variations in *iroN*, *sitA,* and *zinT* promoter activity at the tissue and cellular level in vivo (Fig. 2). These data imply that metal limitation is not evenly distributed within the gut tissue or tissue microcompartment, consistent with reports of differential iron availability in STm-infected spleens (45) and *Staphylococcus aureus* infected tissues (105, 106), and could contribute to the lack of an overt colonization defect for bacterial mutants in vivo.

While the phagocyte-specific transporter Slc11a1/SLC11A1 has assumed a place of prominence in nutritional immunity, here we show that SLC11A2 in IECs also shapes infection outcome via metal deprivation (summarized in *SI* *Appendix*, Fig. S10). Under basal conditions, SLC11A2 localizes to the plasma membrane and endosomes in IECs (75–77, 107). Upon STm infection, SLC11A2 is temporally recruited to SCVs (Fig. 3). In *SLC11A2* KO IECs, we observed enhanced STm proliferation in both the SCV and cytosol (Fig. 4) and decreased *iroN* and *sitA* promoter activity (Fig. 5*B*), specifically in the vacuolar population (Fig. 5*D*). STm employ MntH and EntC to circumvent SLC11A2-driven efflux of iron and manganese from the SCV in cellulo (Fig. 6). By contrast, STm sense equivalent Fe^2+^ levels in the cytosol of *SLC11A2* WT and KO cells (Fig. 5*D*) suggesting that the SLC11A2-dependent restriction of the cytosolic population is not a direct effect of Fe^2+^ availability. Numerous indirect effects of reduced divalent metal ion uptake in *SLC11A2* KO cells have been reported, i.e., altered ferroptosis (108), mitochondria membrane potential (109), Notch signaling (110), lipid peroxidation and lysosomal damage (79), any of which might modulate the cytosolic proliferation of STm. SLC11A2 is one of 32 metal ion transporters from six SLC families—SLC11, SLC30, SLC31, SLC39, SLC40, and SLC41—in mammals (111). While our study unequivocally shows that SLC11A2-mediated sequestration of trace metals contributes to IEC defense against STm in cellulo, whether this and other SLC family members play a similar role in nutritional immunity in the gastrointestinal tract remains an unanswered question, one worthy of attention.

## Materials and Methods

### Bacterial Strains and Plasmids.

*S.* Typhimurium (STm) ST4/74 was the wild-type (WT) strain used in this study (112). WT bacteria constitutively expressing codon-optimized *mCherry* on the chromosome (*glmS::Ptrc-mCherryST::*FRT), STm-mCherry, were created by P22 transduction from STm SL1344 WT *glmS::Ptrc-mCherryST*::Cm (113). The following mutants were created by transduction using P22 lysates prepared from the STm 14028s single gene deletion mutant library (114): ∆*entC*::Kan, ∆*znuA*::Kan, ∆*mntH*::Kan ∆*sitA*::Cm, ∆*zupT*::FRT ∆*znuA*::Kan, ∆*mgtA*::Kan ∆*mgtB*::Cm, and ∆*invA*::FRT ∆*ssaD*::Kan. pCP20 (115) was used for Flp-mediated excision of antibiotic resistance gene cassettes. The transcriptional reporters pMPMA3∆Plac-P*iroN*-*gfpmut3.1,* pMPMA3∆Plac-P*sitA*-*gfpmut3.1,* pMPMA3∆Plac-P*zinT*-*gfpmut3.1*, pMPMA3∆Plac-P*mgtC*-*gfpmut3.1*, pMPMA3∆Plac-P*prgH*-*gfp(LVA)*, and pMPMA3∆Plac-P*ssaG*-*gfp(LVA)* have been described previously (51, 116) and were electroporated into STm-mCherry bacteria. Bacteria stably maintained the transcriptional fusions over the duration of our experiments in cellulo and in vivo (as assessed by replica plating on agar containing no antibiotic and 50 µg/ml carbenicillin). For fluorescence detection of vacuolar and cytosolic STm, pWSK129-P*ssaG*-*mCherryST,* and pWSK129-P*uhpT*-*mCherryST*, respectively, were electroporated into ST4/74 WT pMPMA3∆Plac-P*iroN*-*gfpmut3.1* bacteria. For plasmid-borne complementation, the coding sequences and upstream regulatory regions of *sitA* and *mntH* were cloned into the low copy number plasmid, pWSK29 (117). Oligonucleotides are listed in *SI Appendix*, Table S1. The pWSK29-*entCEBA* plasmid has been described previously (51).

### Mammalian Cell Culture.

*SLC11A2* wild type (WT) and knockout (KO) HCT116 (human colorectal carcinoma epithelial cells) cell pools were generated using CRISPR/Cas9 technology by Synthego. For the WT cell pool, parental cells were electroporated with SpCas9 only and confirmed to be unedited at the *SLC11A2* locus. For the KO cell pool, parental cells were electroporated with SpCas9 and three *SLC11A2*-specific sgRNAs targeting exon 5 (CUAGACUGGGAGUGGUUACU, GUUGCUCUGGAUCCUUCUGU, GACAAUACAUUGCUCACCUU). Gene editing efficiency was >99.9%. Cells were maintained at 37 °C and 5% CO_2_ in McCoy’s 5A (Iwakata and Grace modification, Gibco) medium containing 10% (v/v) heat-inactivated fetal calf serum (FCS; Gibco) and used within 15 passages of receipt.

### Bacterial Infection of Mammalian Cells.

HCT116 *SLC11A2* WT and KO cells were seeded at a density of 8 × 10^4^ cells/well in collagen-coated (rat tail collagen I, Corning) 24-well plates or 3.2 × 10^5^ cells/well in 6-well plates (Thermo Scientific Nunc) in McCoy’s 5A media containing 10% (v/v) FCS. Growth medium was replaced with McCoy’s 5A media containing 1% (v/v) FCS the next day, unless otherwise specified. Bacterial cultures were grown overnight (16 to 20 h) in 2 ml LB-Miller broth (BD Difco) with shaking (220 rpm) at 37 °C, then subcultured 1:33 into 10 ml LB-Miller broth in 125 ml Erlenmeyer flasks. Growth continued at 37 °C for 3.5 h with shaking (220 rpm). Cell monolayers were infected ~48 h postseeding with bacterial subcultures (MOI ~100) for 10 min, then washed thoroughly in Hank’s balanced salt solution to remove noninternalized bacteria. Gentamicin protection assays and CHQ resistance assays (400 µM CHQ) were as described previously (83), except that McCoy’s 5A media containing 1% (v/v) FCS was used for infections, unless otherwise stated. Monolayers were solubilized in 0.2% (w/v) sodium deoxycholate (Sigma) and serial dilutions were plated on LB-Miller agar (BD Difco) for enumeration of colony-forming units (CFUs).

### Statistical Analysis.

All experiments were conducted on at least three separate occasions, unless otherwise indicated. Results are presented as mean ± SD, except for MFI data. Statistical analyses were performed using i) Student’s *t* test, ii) one-way ANOVA with Dunnett’s or Tukey’s multiple comparisons test, or iii) two-way ANOVA with multiple comparison test (GraphPad Prism). A *P*-value of <0.05 was considered significant.

Further detailed descriptions of Materials and Methods for bovine infections, live-cell imaging, immunostaining, fluorescence microscopy, immunoblotting, and ICP/MS analysis are available in the *SI Appendix*.

## Supplementary Material

Appendix 01 (PDF)

## Data Availability

All study data are included in the article and/or *SI Appendix*.
