# Supplementary material for: SLC11A2 withholds divalent metals from Salmonella in the gut epithelium
Source: Proc Natl Acad Sci U S A. 2026 Jun 22;123(26):e2532675123. doi: 10.1073/pnas.2532675123 (PMC13320715; doi:10.1073/pnas.2532675123)
Supplement: Supplementary file 1 — Appendix 01 (PDF) [file pnas.2532675123.sapp.pdf]

Materials and Methods

Bovine infections. The Institutional Animal Care and Use Committee of University of Wisconsin-Madison approved the bovine infection experiment (Protocol number V006249). All experiments were performed in accordance with the PHS "Guide for the Care and Use of Laboratory Animals" in AAALAC-approved animal facilities. Holstein cross-bred calves were obtained from a University of Wisconsin-Madison farm herd. Genetic variations in the bovine *SLC11A1* gene have been described but none are known to negatively affect the expression or targeting (1). Calves were separated from the dam and administered colostrum on the farm and transferred to AAALAC-approved large animal housing facilities within 5 days of birth with housing in individual or grouped isolation rooms. Additional colostrum replacer was administered if determined necessary by measurement of serum total protein to estimate adequate passive transfer of immunity. Calves were fed milk replacer at 10-20% body weight per day with free choice access to water, hay, and calf starter. Selective fecal cultures were performed at least twice weekly and all calves had at least one negative fecal culture for *Salmonella enterica* prior to surgery. For calves with *Salmonella enterica* isolates obtained from feces, a representative isolate was serotyped at the Wisconsin Veterinary Diagnostic Laboratory and no calf was harboring any *Salmonella enterica* serotype B.

In preparation for ligated loop infections, bacteria were grown overnight at 37°C with shaking (225 rpm) in LB broth. Overnight cultures were sub-cultured 1:100 into LB broth and grown for approximately 4 hours at 37°C with shaking (225 rpm). Bacteria were washed twice in PBS and cell concentration was normalized by optical density (OD_{600}). Actual inoculum dose was determined by serial dilution and plating.

At 3-6 weeks of age, calves were placed under general anesthesia with intravenous propofol and maintained with isoflurane inhalant for ligated jejuno-ileal ligated loop surgery as previously described with minor modifications (2). Briefly, calves were positioned in left lateral recumbency and a right flank incision was made. Up to thirty-eight 3- to 6-cm loops were tied in the ileum and terminal jejunum with 1-cm spacers between adjacent loops. Loop lengths were recorded prior to inoculation of 2 ml PBS with ~10^9 CFU of the indicated bacterial strains. Infected intestinal segments were returned

to the abdomen, the incision closed, and the calves were maintained under inhalant anesthesia for the duration of the experiment. Calves were euthanized by intravenous pentobarbital after 2 h or 8 h incubation of infected loops. After euthanasia, the incision was opened, and all loops were excised individually. Following excision, intestinal fluid and tissue samples were harvested and processed. Fluid volume was calculated by excising individual loops and weighing escaped luminal fluid on a sterile Petri dish. Luminal fluid was then transferred to 1 mL PBS to allow for bacterial enumeration. Intestinal tissues were first processed for tissue fixation samples to maintain tissue integrity with the remaining tissue sample washed twice in PBS to remove ingesta and non-adherent bacteria. Washed tissues were then cut in half with one segment processed to quantify tissue-associated bacteria, and the other half was treated with gentamicin (50 µg/mL) for 30 minutes at 37°C to quantify intracellular bacteria. After gentamicin treatment, tissues were washed twice with PBS to remove remaining gentamicin. Samples were homogenized, serially diluted, and plated on the appropriate antibiotics for CFU enumeration.

LysoTracker, immunostaining and fluorescence microscopy. *SLC11A2* WT and KO cells were seeded on collagen-coated Clear FluoroDish Cell Culture Dishes at $1.2x10^5$ cells/dish (35 mm diameter, 23 mm well; World Precision Instruments). Cells were pretreated with 100 nM bafilomycin (Cell Signaling) in McCoy's 5A media containing 1% (v/v) FCS for 1 h at 37°C where indicated. Incubation with 75 nM LysoTracker Red DND-99 (Invitrogen) was for 45 min at 37°C, cells were washed twice in HBSS, then incubated a further 30 min in McCoy's 5A media containing 1% (v/v) FCS prior to live-cell imaging. LysoTracker images were acquired using a Nikon Ti-2E inverted microscope controlled with Nikon NIS-Elements software and outfitted with a Yokogawa CSU-X1 Spinning Disk with perfect-focus and a Photometrics Prime BSI sCMOS camera. Cells were maintained at 37°C with 5% CO_2 in an incubation chamber (OkoLabs) while imaging. LysoTracker channel was obtained using a 60x oil objective (1.4 NA) with a 561nm laser (20% power, 100 ms exposure) and 620/50 nm emission filter. Corresponding brightfield images were also acquired to determine the cell border for data analysis. For analyzing Lysotracker area and mean fluorescence intensity (MFI), ROI of cell outlines were obtained

by freehand selection in ImageJ using a merge of the brightfield channel and LysoTracker channels. Isolated LysoTracker channels were then subjected to rolling ball background subtraction (sliding paraboloid at radius 10) followed by adjustment of min/max display and Otsu thresholding to isolate LysoTracker immunofluorescence signal. Lysotracker fluorescence area (μm^2) and intensity per cell was obtained using the measure function in the ROI manager and limited to the thresholded area. All images within a technical replicate were background subtracted, adjusted and thresholded using the same settings.

For infections, *SLC11A2* WT and KO cells were seeded on collagen-coated, acid-washed glass coverslips (12 mm, #1.5 thickness, Fisher Scientific) in 24-well tissue culture plates. For HCT116 cells infected with STm harboring transcriptional reporters, monolayers were washed once in PBS, then fixed with 2.5% (w/v) paraformaldehyde (PFA) in PBS for 10 min at 37°C. DNA was stained with Hoechst 33342 (Invitrogen) and coverslips were mounted onto glass slides using Mowiol (EMD Millipore) containing 2.5% (w/v) 1,4-diazobicyclo-[2.2.2]-octane (DABCO; Thermo Scientific). For immunodetection of SLC11A2, monolayers were infected with STm-mCherry as described above, washed once in PBS, then fixed in 2.5% PFA for 10 min at 37°C. Cell monolayers were blocked/permeabilized in 10% (v/v) normal goat serum (Gibco)/0.2% (w/v) saponin in PBS (blocking buffer) for 20 min at room temperature (RT), then incubated with rabbit monoclonal anti-SLC11A2/DMT1 (D3V8G, Cell Signaling) diluted 1:100 in blocking buffer for 30-45 min at RT. Coverslips were washed three times with PBS and incubated for a further 30–45 min at RT with goat anti-rabbit Alexa Fluor 488 antibodies (1:400 dilution; Life Technologies) in blocking buffer. After three washes in PBS, cells were incubated with Hoechst 33342 (1:10,000 dilution; Invitrogen) for 1 min before mounting in Mowiol on glass slides. Samples were cured overnight at RT.

Samples were visualized on a Leica Thunder DM4 upright fluorescence microscope and scored for number of bacteria per cell, proportion of GFP-positive bacteria or SLC11A2-positive bacteria, and quantification of GFP fluorescence. To minimize the impact of observer bias (3), samples were blinded prior to analysis. ImageJ software was used to quantify GFP fluorescence (a readout for transcriptional reporter activity) at the individual bacterium level as previously described (4). From

grayscale images of ≥5 randomly selected fields of view on the mCherry channel (representative of all bacteria (*glmS*::P*trc-mCherryST*), vacuolar bacteria (pWSK129-P*ssaG-mCherryST)* or cytosolic bacteria (pWSK129-P*uhpT-mCherryST*)), 1-5 well-defined bacteria per cell were arbitrarily chosen, manually outlined, and converted into a binary image. The MFI of each bacterium was then determined by transferring the mask from the mCherry channel to the corresponding GFP channel image.

Bovine intestinal tissue fixation and staining was as described previously with minor modifications (5). Bovine tissue samples were transferred to tissue cassettes and placed into 10% buffered formalin for 24 h. Samples were then floated into 20% (w/v) sucrose with 0.05% (w/v) sodium azide and stored at 4°C until use. Tissue samples were submitted to the University of Wisconsin-Madison Translational Research Initiatives in Pathology (TRIP) laboratory for tissue embedding, freezing, and tissue sectioning (10 μm). For tissues infected with STm-mCherry harboring GFP transcriptional reporters, sections were rehydrated in PBS for 5 min, blocked with 2% normal donkey serum (EMD Millipore), 1% bovine serum albumin (BSA CELLect, MP Biomedicals), 0.1% Triton X-100 (MP Biomedicals), and 0.05% Tween 20 (Calbiochem) in PBS (NDS/TX100/Tween/PBS) for 30 min at RT. For immunostaining, sections were rehydrated with PBS for 5 min, followed by permeabilization and blocking with NDS/TX100/Tween/PBS for 45 min at RT. Sections were incubated overnight at 4°C in a humidified chamber with rabbit polyclonal anti-human SLC11A1 (1:100 dilution, NBP1-87809, NovusBio) (antibody was developed against a human SLC11A1 peptide sequence that shares 87% identity with bovine SLC11A1) or rabbit monoclonal anti-SLC11A2 (1:100 dilution, D3V8G, Cell Signaling Technology) in 1% BSA, 0.1% TX100, and 0.05% Tween 20 in PBS, followed by 3 washes for 5 min each in 0.05% Tween 20 in PBS (PBST). Donkey anti-rabbit Alexa Fluor 488 and phalloidin Alexa Fluor 647 (Invitrogen) were diluted 1:400 in 0.1% Triton X-100 in 0.05% Tween 20 in PBS and incubated for 1 h at RT. Sections were washed 3 times for 5 min each in PBST, then covered with ProLong Gold antifade reagent with DAPI (Life Technologies), followed by a glass coverslip (Corning). Samples were cured overnight at RT. Slides were viewed at 63x or 100x on a Leica Thunder DM4 upright fluorescence microscope. Images were processed using Adobe Photoshop 2024.

Immunoblotting. Monolayers in 6-well plates were washed twice with PBS prior to lysis in boiling 1.5x SDS-PAGE sample buffer. Proteins were separated by SDS-PAGE and subsequently transferred to 0.2 µm nitrocellulose membranes (Amersham). Membranes were blocked at room temperature for 1-2 h with Tris-buffered saline (TBS) containing 5% (w/v) skim milk powder and 0.1% (v/v) Tween-20 (TBST-milk) for SLC11A2, SLC40A1, CD71 and COX IV, or TBST with 3% (w/v) BSA (Millipore) (TBST-BSA) for LCN2, SLC39A14, ferritin heavy chain (FTH1) and ferritin light chain (FTL). Membranes were then incubated overnight at 4°C with the following primary antibodies in TSBT-milk (SLC11A2, SLC40A1, CD71, COX IV) or TBST-BSA (LCN2, SLC39A14, FTH1, FTL): rabbit monoclonal anti-SLC11A2 (D3V8G, 1:1,000 dilution, Cell Signaling), rabbit polyclonal anti-SLC40A1/FPN1 (26601-1-AP, 1:4,000 dilution, Proteintech), rabbit monoclonal anti-CD71 (D7G9X, 1:5,000 dilution, Cell Signaling), rabbit monoclonal anti-LCN2 (D4M8L, 1:1,000 dilution, Cell Signaling), rabbit monoclonal anti-SLC39A14/ZIP14 (E3H7D, 1:1,000 dilution, Cell Signaling), rabbit monoclonal anti-FTH1 (D1D4, 1:1,000 dilution, Cell Signaling), rabbit monoclonal anti-FTL (F4T8H, 1:1,000 dilution, Cell Signaling) or rabbit monoclonal anti-COX IV (3E11, 1:5,000 dilution, Cell Signaling). Membranes were then incubated with anti-rabbit IgG horseradish peroxidase (HRP)-conjugated secondary antibodies (1:10,000 dilution, Cell Signaling) in TBST-milk for 1 h at RT, followed by Supersignal West Femto Max Sensitivity ECL Substrate (Thermo). Chemiluminescence was detected using a GE Healthcare AI600 imager. ImageJ software was used to quantify protein band intensity relative to the COX IV loading control.

Total Metals Quantitation. *SLC11A2* WT and KO cells were seeded in 4x15 cm tissue culture treated dishes (Nunc) at $1x10^7$ cells per dish. Growth media was changed to McCoy's containing 1% FCS the next day, then mock-infected or STm-infected for 8 h the following day. Monolayers were washed with 30 ml tissue culture grade phosphate buffered saline without calcium and magnesium (PBS^{--}, Corning), then scraped into 10 ml PBS^{--}, collected and pooled in a metal-free 50 ml centrifuge tube (VWR) and centrifuged at 400xg for 5 min. The supernatant was removed, the cell pellet resuspended

in 10 ml PBS^{--}, then transferred to a metal-free 15 ml centrifuge tube (VWR) and centrifuged again. The supernatant was removed and the wet weight of the cell pellet recorded. Samples were digested in 600 µL 70% Optima-grade nitric acid at 65°C overnight, then diluted with UltraPure water to 20% nitric acid for analysis. Elemental quantification was conducted using an Agilent 7700 ICP-MS attached to an ASX-560 autosampler. The settings for analysis were cell entrance = −40 V, cell exit = −60 V, plate bias = −60 V, OctP bias = −18 V, and helium flow = 4.5 ml/min. Optimal voltages for extract 2, omega bias, omega lens, OctP RF, and deflect were empirically determined. Calibration curves for elements were generated using ARISTAR ICP standard mix. Samples were introduced by peristaltic pump with 0.5-mm-internal-diameter tubing through a MicroMist borosilicate glass nebulizer. They were initially taken up at 0.5 rps for 30 seconds, followed by 30 seconds at 0.1 rps to stabilize the signal. Spectrum mode analysis was performed at 0.1 rps, collecting three points across each peak and conducting three replicates of 100 sweeps for each element. The sampling probe and tubing were rinsed with 2% nitric acid for 30 seconds at 0.5 rps between each sample. Data were acquired and analyzed using Agilent MassHunter workstation software version A.01.02. The concentration of each metal (in ppb) was normalized to that of ^{34}S (in ppb) in each sample.

References

1. A. Holder, *et al.*, Analysis of Genetic Variation in the Bovine SLC11A1 Gene, Its Influence on the Expression of NRAMP1 and Potential Association With Resistance to Bovine Tuberculosis. *Front Microbiol* **11**, 1420 (2020).

2. J. R. Elfenbein, *et al.*, Novel determinants of intestinal colonization of *Salmonella enterica* serotype typhimurium identified in bovine enteric infection. *Infect Immun* **81**, 4311–4320 (2013).

3. A. P.-T. Jost, J. C. Waters, Designing a rigorous microscopy experiment: Validating methods and avoiding bias. *J Cell Biol* **218**, 1452–1466 (2019).

4. T. R. Powers, *et al.*, Intracellular niche-specific profiling reveals transcriptional adaptations required for the cytosolic lifestyle of *Salmonella enterica*. *PLOS Pathogens* **17**, e1009280 (2021).

5. R. C. Laughlin, *et al.*, Spatial segregation of virulence gene expression during acute enteric infection with *Salmonella enterica* serovar Typhimurium. *mBio* **5**, e00946-00913 (2014).

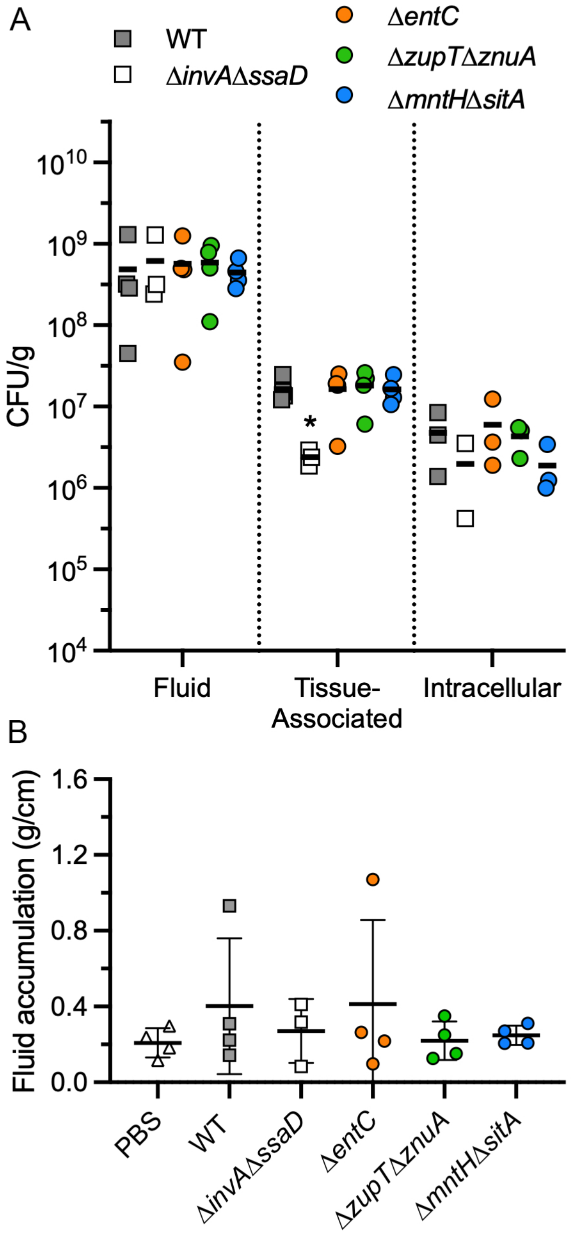

Figure S1: Colonization and fluid accumulation at 2 h post-inoculation. Ligated ileal loops were inoculated with PBS, wild-type (WT) bacteria, or the indicated deletion mutant (~10^9 CFU) for 2 h. (A) CFU from washed intestinal tissue (total tissue-associated bacteria), gentamicin-treated intestinal tissue (intracellular) and fluid were enumerated by serial dilution and plating on LB agar. CFU were normalized to the tissue or fluid weight. (B) Fluid weight was normalized to loop length. Each symbol represents data from one loop from one calf. *$p<0.05$, significantly different from ST4/74 WT bacteria, ANOVA with Dunnett's multiple comparisons.

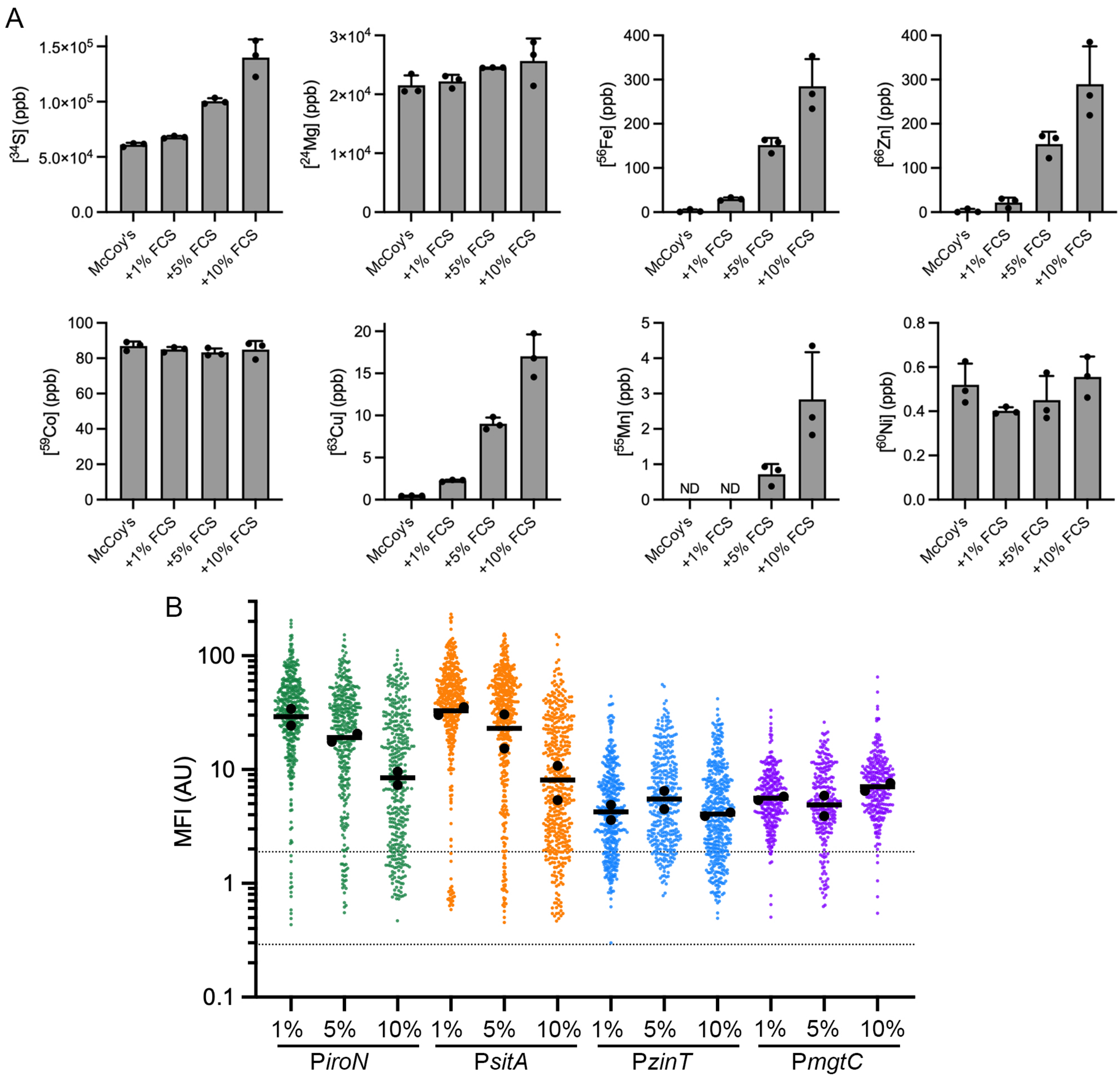

Figure S2: Serum concentration in growth media affects extracellular and intracellular metal levels. (A) Total levels of naturally occuring abundant isotopes of ^{34}S, ^{24}Mg, ^{56}Fe, ^{66}Zn, ^{59}Co, ^{63}Cu, ^{55}Mn and ^{60}Ni in McCoy's 5A media (no FCS) and McCoy's 5A media supplemented with 1%, 5% or 10% (v/v) FCS were quantified by ICP-MS analysis. Three independent lot numbers of media were tested. ND, none detected. (B) HCT116 WT cells in McCoy's media containing 1%, 5% or 10% (v/v) FCS were infected with STm-mCherry harboring fluorescent transcriptional reporters. Monolayers were fixed at 8 h p.i. and stained with Hoecsht 33342 to label DNA. The MFI of GFP signal was quantified by fluorescence microscopy and ImageJ analysis. AU, arbitrary units. Small dots represent individual bacteria; large dots the geometric mean of each experiment; horizontal solid lines the average of two independent experiments.

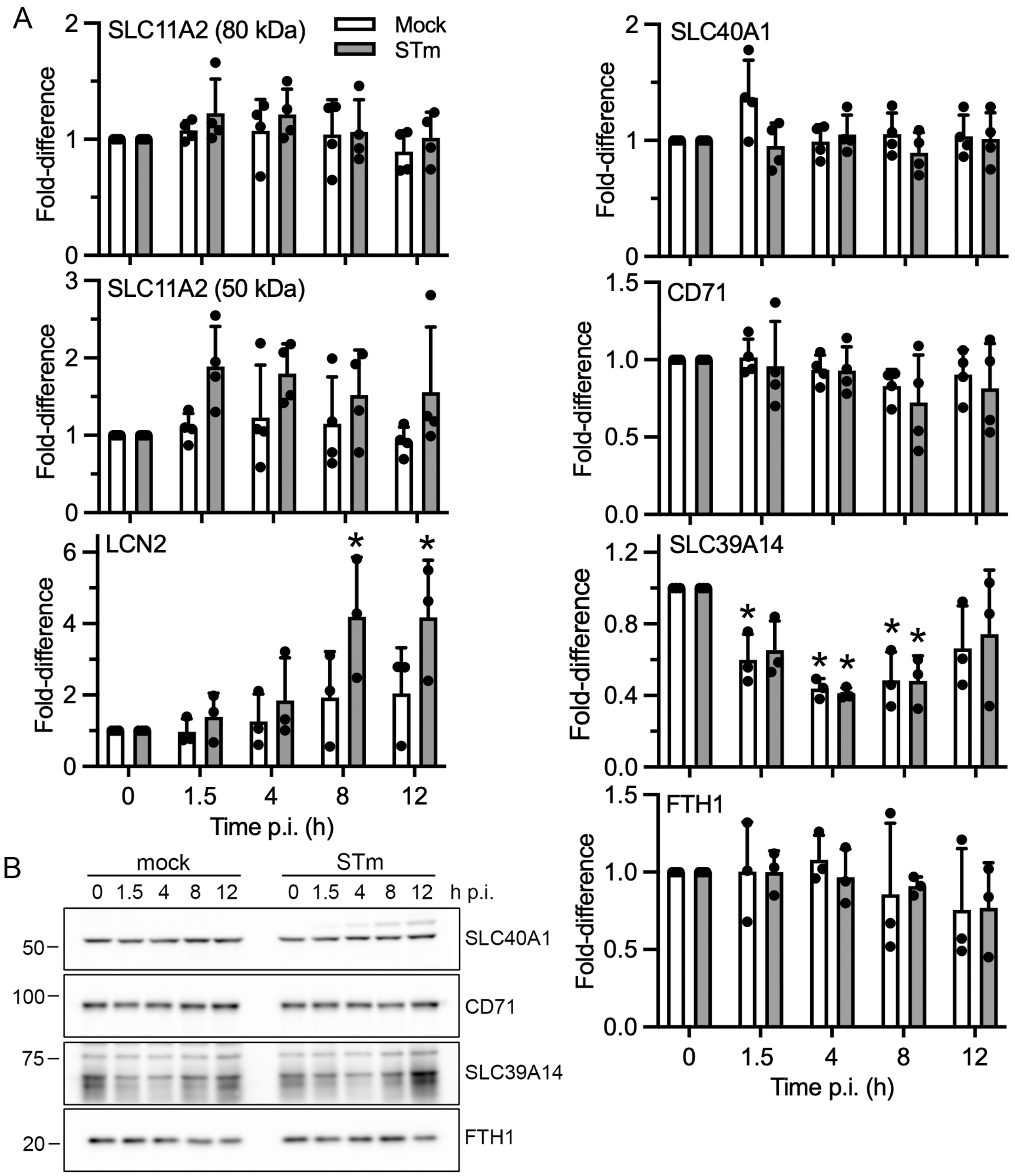

Figure S3: Quantification of immunoblots from mock- and STm-infected HCT116 lysates. HCT116 whole cell lysates were collected at the indicated times post-infection (p.i.) (McCoy's media containing 1% (v/v) FCS). Proteins were separated by SDS-PAGE and subject to immunoblotting with antibodies against SLC11A2 (NRAMP2), lipocalin-2 (LCN2), SLC40A1 (FPN1), CD71 (Tfr1), SLC39A14 (ZIP14), ferritin heavy chain (FTH1) and cytochrome c oxidase IV (COX IV). (A) Bands were quantified by densitometry (ImageJ) and normalized to COX IV (loading control) for each sample, then expressed as a fold-change compared to t_0. The SLC11A2 80 kDa and 50 kDa bands, representing different isoforms, were quantified separately. Mean ± SD from 3-4 independent experiments. Asterisks indicate data significantly different from t_0, $p<0.05$, ANOVA with Dunnett's multiple comparisons test. (B) Representative immunoblots for SLC40A1, CD71, SLC39A14 and FTH1 are shown. Molecular mass markers are shown on the left.

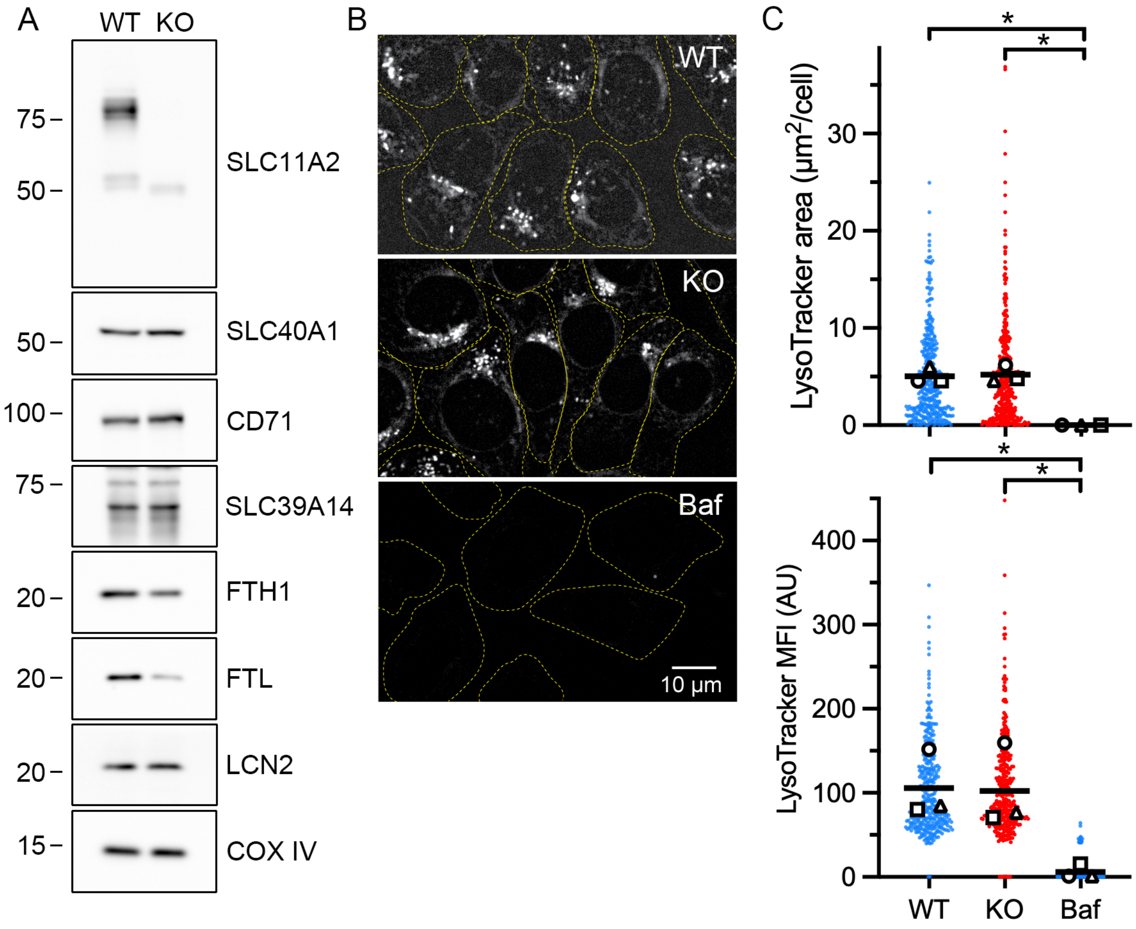

Figure S4: Characterization of *SLC11A2* knockout (KO) cells. (A) HCT116 *SLC11A2* WT and KO whole-cell lysates (McCoy's media containing 1% (v/v) FCS) were probed with antibodies against SLC11A2, SLC40A1 (FPN1), CD71 (TfR1), SLC39A14 (ZIP14), ferritin heavy chain (FTH1), ferritin light chain (FTL), lipocalin-2 (LCN2) and COX IV (loading control). Molecular mass markers are indicated on the left. Representative immunoblots are shown. (B) Representative fluorescence microscopy images of LysoTracker Red DND-99 from *SLC11A2* WT, KO and bafilomycin (Baf)-treated WT cells. Dotted lines (yellow) indicate each cell border. (C) Analysis of LysoTracker Red DND-99 area (upper panel) and mean fluorescence intensity (MFI) in arbitrary units (AU) per cell (lower panel). Small dots represent individual cells; large open symbols indicate the mean of each experiment; horizontal bars indicate the mean of three independent experiments. A total of 353 (WT), 344 (KO) and 113 (Baf-treated) cells were analyzed. *p<0.05, ANOVA with Tukey's multiple comparisons test.

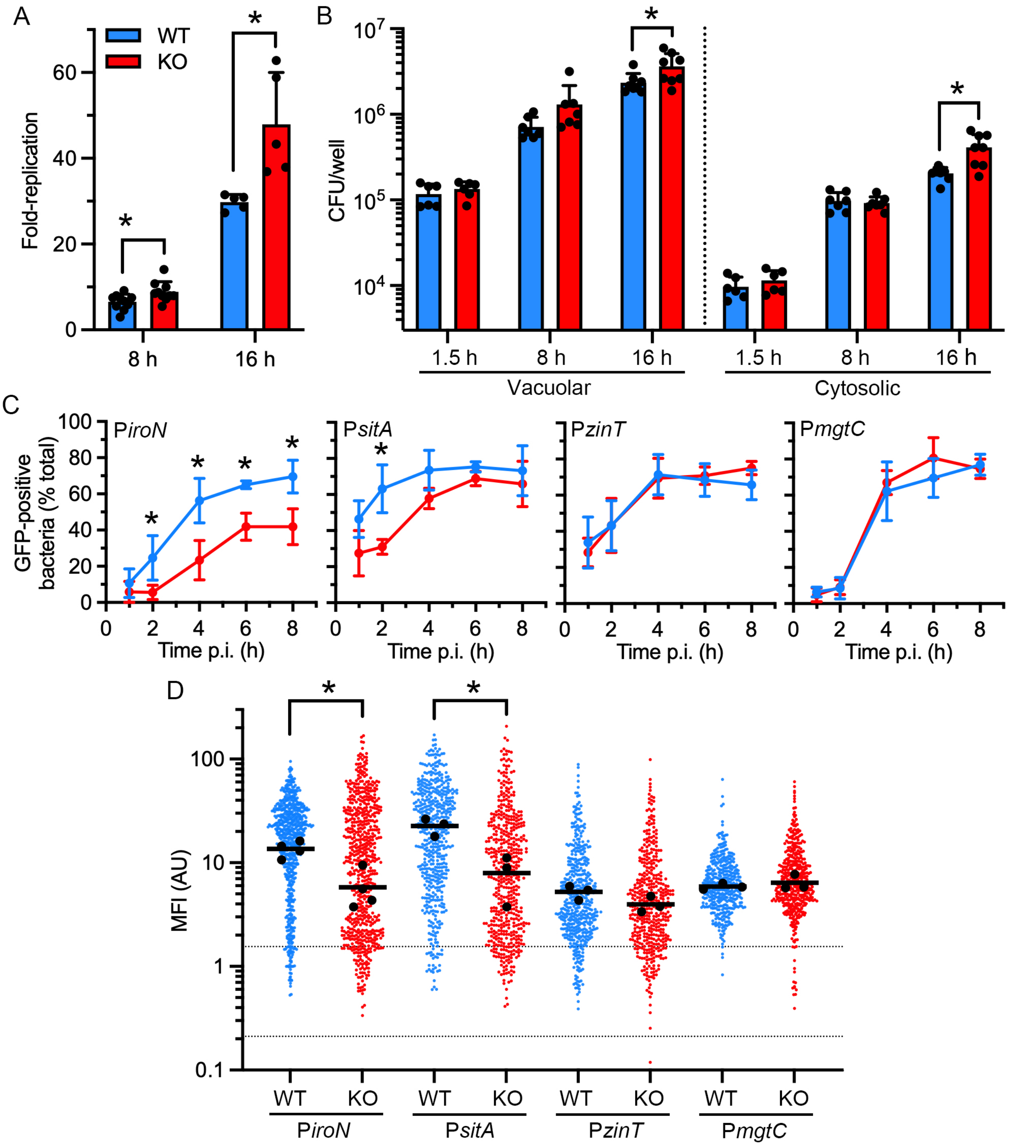

Figure S5: Bacterial replication and fluorescent reporter activation *in cellulo* (McCoy's media with 5% (v/v) FCS). (A) HCT116 *SLC11A2* WT and KO cells were infected with STm and the number of internalized bacteria at 1 h, 8 h and 16 h p.i. was quantified by gentamicin protection assay. Fold-replication was determined by dividing CFUs at 8 h or 16 h p.i. by those at 1 h p.i. Mean ± SD, n ≥ 5 independent experiments. (B) Vacuolar and cytosolic bacteria were determined by CHQ resistance assay in conjunction with a gentamicin protection assay. CHQ-resistant bacteria are cytosolic, CHQ-sensitive bacteria are vacuolar. Mean ± SD, n ≥ 6 independent experiments. (C) WT and KO cells were infected with STm-mCherry harboring fluorescent transcriptional reporters. At the indicated times, monolayers were fixed and stained with Hoechst 33342 to label DNA. The number of GFP-positive bacteria was scored by fluorescence microscopy. Mean ± SD, n ≥ 3 independent experiments. (D) The mean fluorescence intensity (MFI) of GFP signal at 8 h p.i. was quantified by fluorescence microscopy and Image J analysis. AU, arbitrary units. Small dots represent individual bacteria; large dots the geometric mean of each experiment; horizontal solid lines the average of 3-4 independent experiments. *p<0.05, Student's t-test.

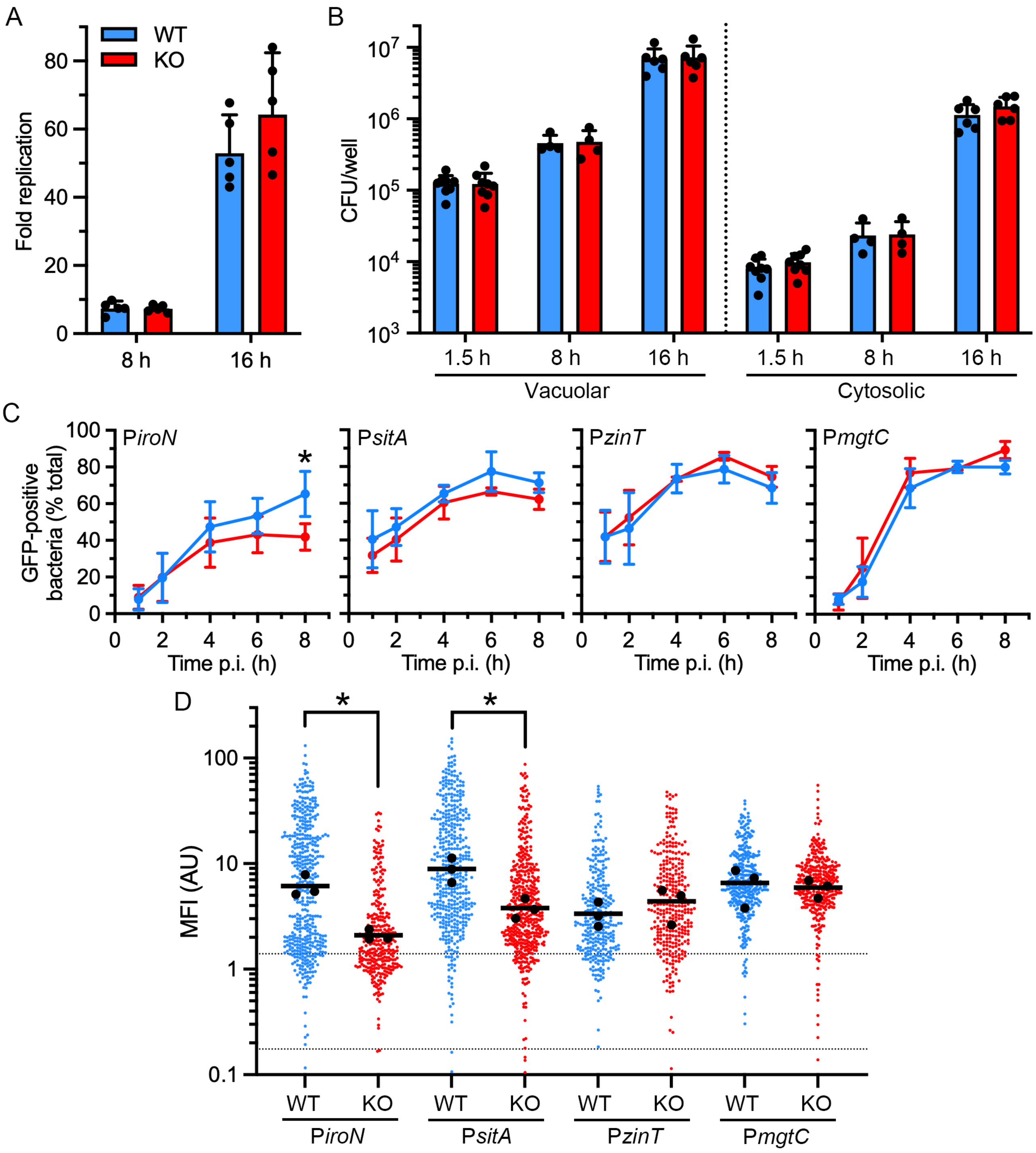

Figure S6: Bacterial replication and fluorescent reporter activation *in cellulo* (McCoy's media with 10% (v/v) FCS). (A) HCT116 *SLC11A2* WT and KO cells were infected with STm and the number of internalized bacteria at 1 h, 8 h and 16 h p.i. was quantified by gentamicin protection assay. Fold-replication was determined by dividing CFUs at 8 h or 16 h p.i. by those at 1 h p.i. Mean ± SD, n = 5 independent experiments. (B) Vacuolar and cytosolic bacteria were determined by CHQ resistance assay in conjunction with a gentamicin protection assay. CHQ-resistant bacteria are cytosolic, CHQ-sensitive bacteria are vacuolar. Mean ± SD, n ≥ 4 independent experiments. (C) WT and KO cells were infected with STm-mCherry harboring fluorescent transcriptional reporters. At the indicated times, monolayers were fixed and stained with Hoechst 33342 to label DNA. The number of GFP-positive bacteria was scored by fluorescence microscopy. Mean ± SD, n ≥ 3 independent experiments. (D) The mean fluorescence intensity (MFI) of GFP signal at 8 h p.i. was quantified by fluorescence microscopy and Image J analysis. AU, arbitrary units. Small dots represent individual bacteria; large dots the geometric mean of each experiment; horizontal solid lines the average of 3 independent experiments. *p<0.05, Student's t-test.

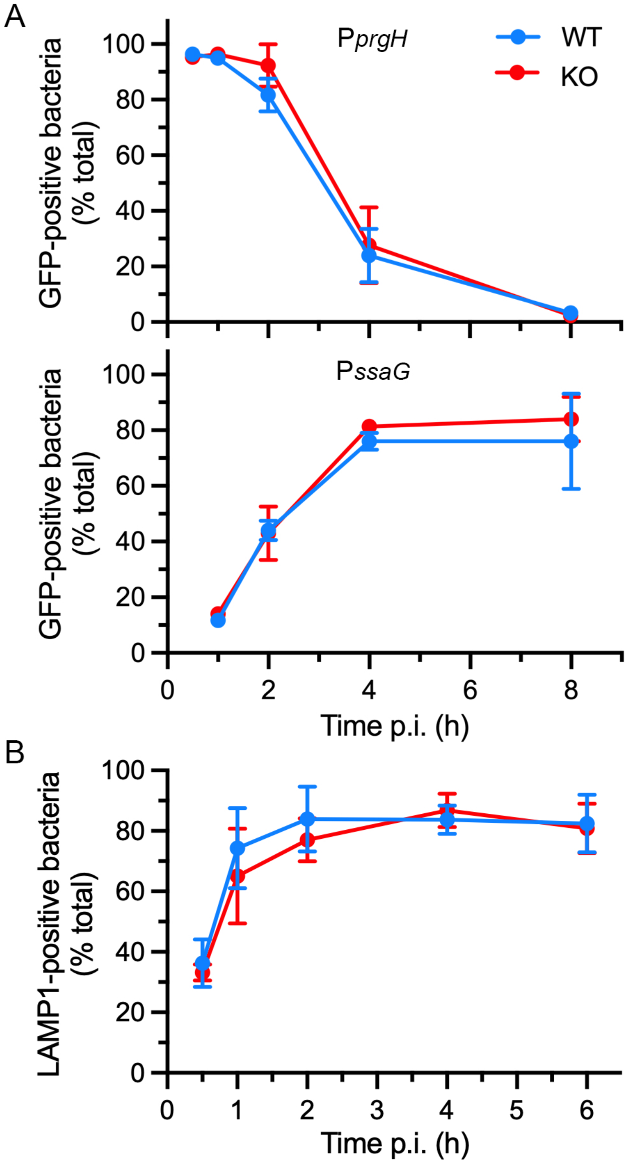

Figure S7: Intracellular virulence gene expression and *Salmonella*-containing vacuole trafficking. (A) HCT116 *SLC11A2* WT and KO cells were infected with STm-mCherry harboring P*prgH-gfp(lva)* or P*ssaG-gfp(lva)* transcriptional reporters (McCoy's media containing 1% (v/v) FCS). At the times indicated, monolayers were fixed and the number of GFP-positive bacteria scored by fluorescence microscopy. (B) WT and KO cells were infected with STm-mCherry (McCoy's media containing 1% (v/v) FCS). At the times indicated, monolayers were fixed and immunostained with anti-LAMP1 antibodies. The proportion of bacteria in LAMP1-positive vacuoles was scored by fluorescence microscopy. Mean ± SD from three independent experiments.

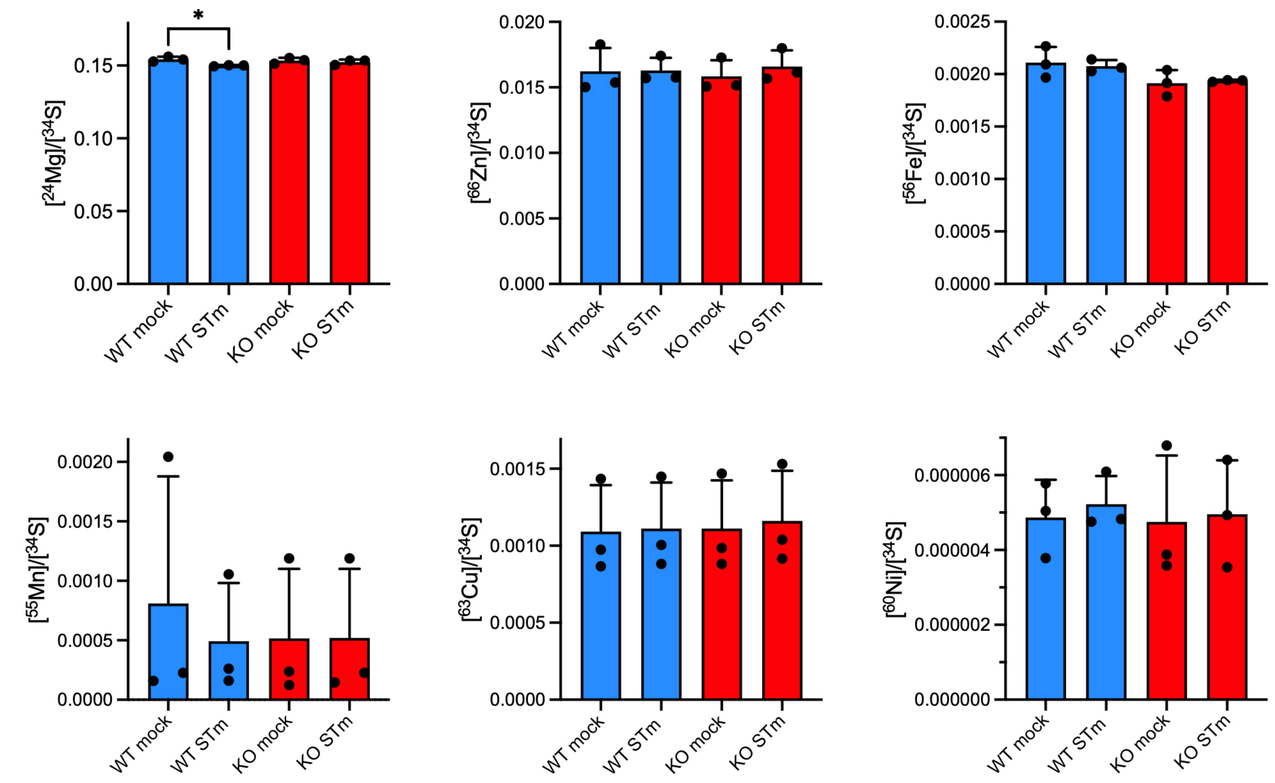

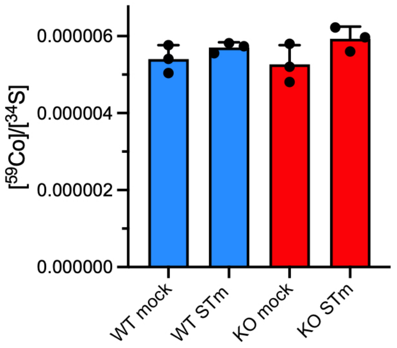

Figure S8: ICP-MS characterization of HCT116 *SLC11A2* WT and KO cells. HCT116 cells were mock- or STm-infected (McCoy's media containing 1% (v/v) FCS) and collected at 8 h p.i. Total cellular levels of naturally occuring abundant isotopes of ^{24}Mg, ^{66}Zn, ^{56}Fe, ^{55}Mn, ^{63}Cu, ^{60}Ni, ^{59}Co and ^{34}S were quantified by ICP-MS analysis. The concentration of each metal (in ppb) was normalized to that of ^{34}S (in ppb) in each sample. Results are the mean ± SD of 3 independent experiments. *$p<0.05$, ANOVA with Tukey's multiple comparisons test.

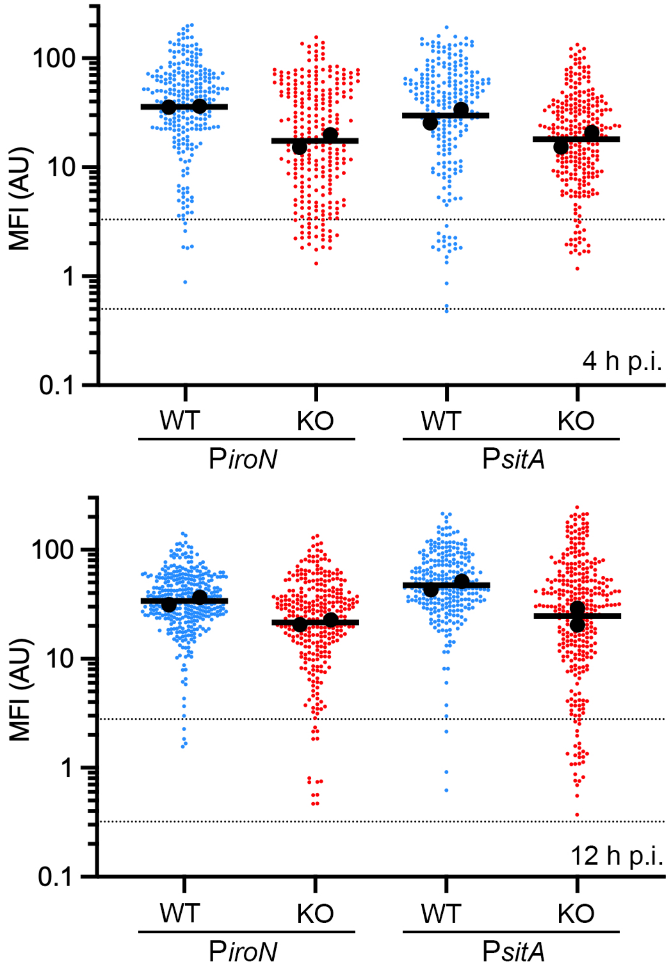

Figure S9: SLC11A2 withholds iron and manganese from intracellular *Salmonella*. HCT116 *SLC11A2* WT and KO cells seeded on glass coverslips were infected with STm-mCherry harboring P*iroN-gfpmut3* or P*sitA-gfpmut3* transcriptional reporters (McCoy's media containing 1% (v/v) FCS). At 4 h p.i. and 12 h p.i., monolayers were fixed and stained with Hoechst 33342 to label DNA. The mean fluorescence intensity (MFI) of GFP signal was quantified by fluorescence microscopy and ImageJ analysis. Acquisition parameters (exposure time and gain) were the same for both reporters at each timepoint. AU, arbitrary units. Small dots represent individual bacteria; large dots the geometric mean of each experiment; horizontal solid lines the average of two independent experiments. The dashed lines indicate the range of background fluorescence in the GFP channel for STm-mCherry (no reporter plasmid).

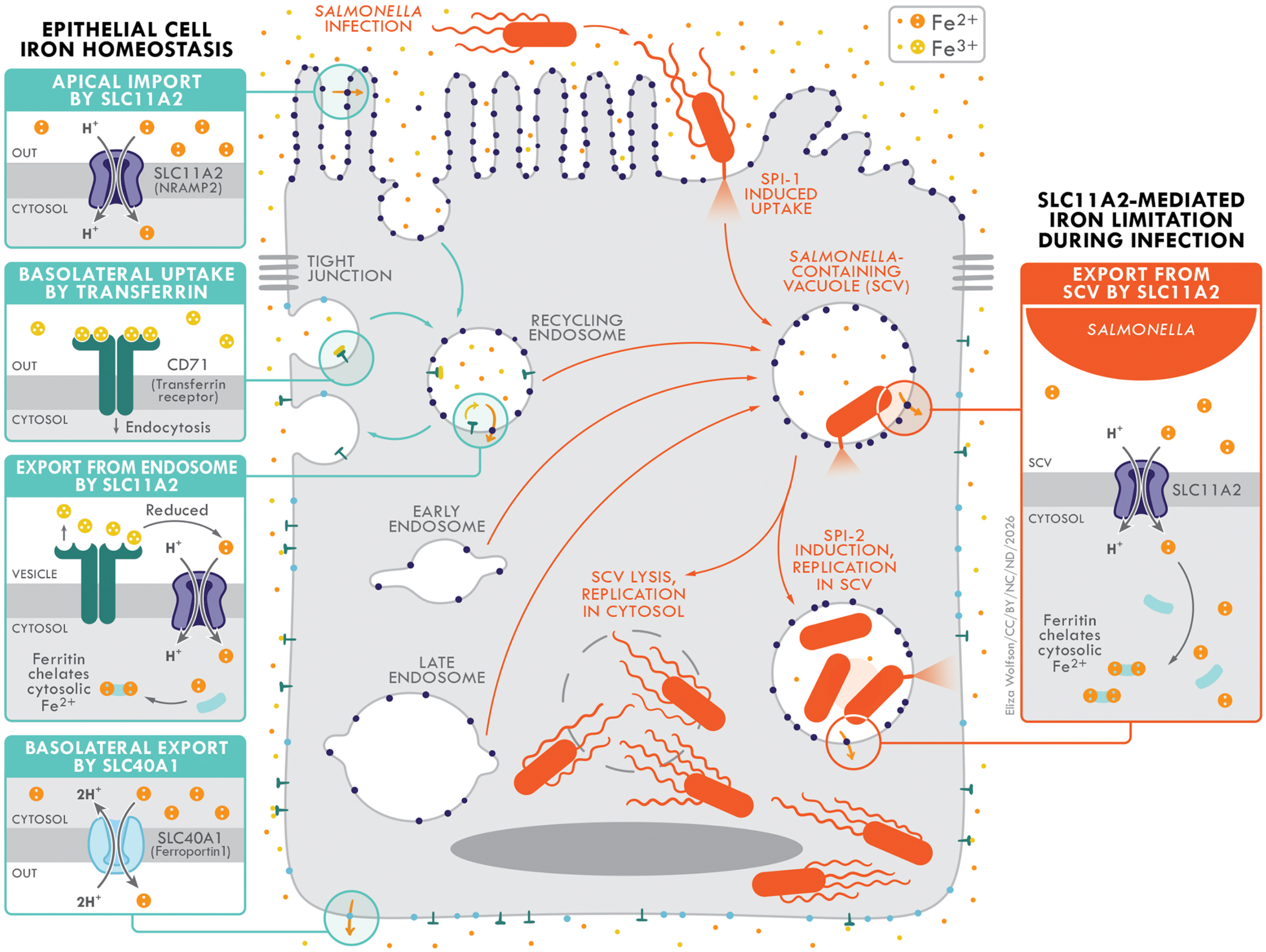

Figure S10: Model depicting SLC11A2-mediated metal restriction during *Salmonella* infection of IECs. During cellular iron homeostasis, iron uptake, storage and efflux are balanced. Non-transferrin bound iron is reduced to Fe^{2+} by ferrireductases at the apical plasma membrane. Fe^{2+} is then imported by SLC11A2 (also known as NRAMP2 or DMT1), a H^+/divalent metal symporter with broad substrate specificity. Transferrin bound-iron (Fe^{3+}) is endocytosed at the basolateral surface by CD71 (transferrin receptor 1, TfR1). Fe^{3+} dissociates from transferrin in endosomes, is reduced to Fe^{2+}, and then exported to the cytosol by SLC11A2. Cytosolic Fe^{2+} can bind to storage proteins such as ferritin, be delivered to mitochondria for haem and iron-sulfur biosynthesis (not shown), be exported by SLC40A1 (ferroportin 1, FPN1), an electroneutral H^+/Fe^{2+} antiporter on the basolateral surface, or form the labile iron pool. During *Salmonella* infection, SLC11A2 is recruited to the *Salmonella*-containing vacuole (SCV) membrane and exports Fe^{2+} and Mn^{2+}, reducing the availability of these metals to intravacuolar bacteria, and limits their proliferation. SLC11A2 also restricts the proliferation of cytosolic *Salmonella* by an undefined mechanism. *Salmonella* counters the actions of SLC11A2 via the Fe^{2+}/Mn^{2+} transporter, MntH, and enterobactin (EntC), a high-affinity iron binding siderophore.

Table S1: Oligonucleotides used in this study

**Name**	**Sequence (5' to 3")**	**Plasmid**
sitAcomp_XhoF	CCGCTCGAGGACCTGCCCAACGCATAATC	pWSK29-*sitA*
sitAcomp_HindR	CCCAAGCTTGAC**TCA**TTGTTGACTCCTCAG	pWSK29-*sitA*
mntHcomp_XhoF	CCGCTCGAGAACAAGTAACTGAATGACGT	pWSK29-*mntH*
mntHcomp_HindR	CCCAAGCT**TTA**TGACAACCCCATCACCG	pWSK29-*mntH*
SalI-PuhpT-F	ACGCGTCGACAGTGCTCGATACCTGGCACTGGAG	pWSK129-P*uhpT*-*mCherry*ST
SalI-PssaG-F	ACGCGTCGACTGGTAGTTTGGGACTACAGCCTCA	pWSK129-P*ssaG*-*mCherry*ST
EcoRI-mCherryST-R	GGAATTC**CTA**TTTGTACAGTTCGTCCATACC	pWSK129-P*uhpT*-*mCherry*ST, pWSK129-P*ssaG-mCherry*ST

Engineered restriction sites are underlined. Stop codons are in **bold**.
